# Factors Associated With Inpatient Subspecialty Consultation Patterns Among Pediatric Hospitalists

**DOI:** 10.1001/jamanetworkopen.2023.2648

**Published:** 2023-03-13

**Authors:** Andrew S. Kern-Goldberger, Evan M. Dalton, Irit R. Rasooly, Morgan Congdon, Deepthi Gunturi, Lezhou Wu, Yun Li, Jeffrey S. Gerber, Christopher P. Bonafide

**Affiliations:** 1 Section of Pediatric Hospital Medicine, Children’s Hospital of Philadelphia, Philadelphia, Pennsylvania; 2 Department of Pediatric Hospital Medicine, Cleveland Clinic, Cleveland, Ohio; 3 Center for Value-Based Care Research, Cleveland Clinic, Cleveland, Ohio; 4 Department of Quantitative Health Sciences, Cleveland Clinic, Cleveland, Ohio.; 5 Clinical Futures, Children’s Hospital of Philadelphia, Philadelphia, Pennsylvania; 6 Department of Biomedical and Health Informatics, Children’s Hospital of Philadelphia, Philadelphia, Pennsylvania; 7 Department of Pediatrics, Perelman School of Medicine at the University of Pennsylvania, Philadelphia; 8 Department of Biostatistics, Epidemiology and Informatics, Perelman School of Medicine at the University of Pennsylvania, Philadelphia

## Abstract

**Importance:**

Subspecialty consultation is a frequent, consequential practice in the pediatric inpatient setting. Little is known about factors affecting consultation practices.

**Objectives:**

To identify patient, physician, admission, and systems characteristics that are independently associated with subspecialty consultation among pediatric hospitalists at the patient-day level and to describe variation in consultation utilization among pediatric hospitalist physicians.

**Design, Setting, and Participants:**

This retrospective cohort study of hospitalized children used electronic health record data from October 1, 2015, through December 31, 2020, combined with a cross-sectional physician survey completed between March 3 and April 11, 2021. The study was conducted at a freestanding quaternary children’s hospital. Physician survey participants were active pediatric hospitalists. The patient cohort included children hospitalized with 1 of 15 common conditions, excluding patients with complex chronic conditions, intensive care unit stay, or 30-day readmission for the same condition. Data were analyzed from June 2021 to January 2023.

**Exposures:**

Patient (sex, age, race and ethnicity), admission (condition, insurance, year), physician (experience, anxiety due to uncertainty, gender), and systems (hospitalization day, day of week, inpatient team, and prior consultation) characteristics.

**Main Outcomes and Measures:**

The primary outcome was receipt of inpatient consultation on each patient-day. Risk-adjusted consultation rates, expressed as number of patient-days consulting per 100, were compared between physicians.

**Results:**

We evaluated 15 922 patient-days attributed to 92 surveyed physicians (68 [74%] women; 74 [80%] with ≥3 years’ attending experience) caring for 7283 unique patients (3955 [54%] male patients; 3450 [47%] non-Hispanic Black and 2174 [30%] non-Hispanic White patients; median [IQR] age, 2.5 ([0.9-6.5] years). Odds of consultation were higher among patients with private insurance compared with those with Medicaid (adjusted odds ratio [aOR], 1.19 [95% CI, 1.01-1.42]; *P* = .04) and physicians with 0 to 2 years of experience vs those with 3 to 10 years of experience (aOR, 1.42 [95% CI, 1.08-1.88]; *P* = .01). Hospitalist anxiety due to uncertainty was not associated with consultation. Among patient-days with at least 1 consultation, non-Hispanic White race and ethnicity was associated with higher odds of multiple consultations vs non-Hispanic Black race and ethnicity (aOR, 2.23 [95% CI, 1.20-4.13]; *P* = .01). Risk-adjusted physician consultation rates were 2.1 times higher in the top quartile of consultation use (mean [SD], 9.8 [2.0] patient-days consulting per 100) compared with the bottom quartile (mean [SD], 4.7 [0.8] patient-days consulting per 100; *P* < .001).

**Conclusions and Relevance:**

In this cohort study, consultation use varied widely and was associated with patient, physician, and systems factors. These findings offer specific targets for improving value and equity in pediatric inpatient consultation.

## Key Points

**Question:**

Which factors are associated with subspecialty consultation in the pediatric inpatient setting, and how does consultation vary among pediatric hospitalists?

**Findings:**

In this cohort study of 7283 hospitalized children cared for by 92 surveyed pediatric hospitalist physicians, private insurance and 0 to 2 years of attending physician experience were associated with greater odds of consultation. At the physician level, risk-adjusted consultation use was 2.1 times higher in the top quartile of consultation use compared with the bottom quartile.

**Meaning:**

These findings suggest that consultation use varies significantly among pediatric hospitalists and is not solely related to clinical need.

## Teaser

This cohort study identifies patient, physician, admission, and systems characteristics that are independently associated with subspecialty consultation among pediatric hospitalists.

## Introduction

Consultation of subspecialty physicians is a common hospital practice. Consulting subspecialists often contribute expertise vital to informing diagnosis, guiding management, or establishing outpatient follow-up. However, in some situations, consultation has unintended negative consequences, including unnecessary testing and outpatient follow-up,^1^ conflicting subspecialty recommendations,^2^ delays in care,^3,4,5^ patient and family dissatisfaction,^6^ and miscommunication between teams.^7,8^ A retrospective cohort study of more than 700 000 admissions among adult Medicare beneficiaries found higher health care resource use when consultations were performed, without mortality benefit.^9^ Studies of adult patients suggest significant subjectivity and variability in the extent of consultation utilization within and across hospitals.^2,5^

While consultation practice patterns in adult populations have been characterized,^2,5,9,10,11^ our understanding of how and why pediatric hospitalists utilize consultations is limited. One descriptive study^12^ found highly variable consultation utilization in 200 hospitalized children with uncertain diagnoses. Studies focused on a single condition (Kawasaki disease) highlighted self-reported variation in consultation among pediatric hospitalists^13^ as well as modest cost and utilization differences among hospitals with different subspecialty care models.^14^

Particularly with pediatric hospital medicine emerging as a board-certified subspecialty requiring proficiency in a broad scope of acute care practice,^15^ it is imperative to elucidate the factors driving variation in pediatric inpatient consultation.^16^ Among a cohort of hospitalized children with 15 distinct, common clinical conditions, our objectives were to (1) identify patient, physician, admission, and systems characteristics that are independently associated with subspecialty consultation among pediatric hospitalists at the patient-day level and (2) describe variation in consultation utilization among pediatric hospitalists.

## Methods

### Study Design and Setting

This was a retrospective cohort study using electronic health record (EHR) data and findings from a cross-sectional survey of pediatric hospitalist physicians at an urban, quaternary children’s hospital with approximately 29 000 annual admissions. Pediatric residents and advanced practice practitioners (APPs) place orders (including consultation orders) for patients; however, plans of care are generally discussed with the supervising attending physician. This study was approved by the Children’s Hospital of Philadelphia institutional review board. The institutional review board granted waivers of parental consent and assent for patients in this minimal risk, retrospective study; the elements of informed consent were included in the physician survey and physicians provided informed consent through survey completion, with a waiver of documentation of consent for physicians granted by the institutional review board. We followed the Strengthening the Reporting of Observational Studies in Epidemiology (STROBE) guideline for reporting of cohort studies.

### Participants and Data Collection

#### Physician Survey

We distributed an electronic REDCap^17^ questionnaire to the 102 active pediatric hospitalists between March 3 and April 11, 2021. The questionnaire included questions about physician demographic characteristics, years of attending clinical experience, and the 15-item revised Physicians’ Reactions to Uncertainty (PRU) scales.^18,19,20^ The instrument uses validated scales to measure physician tolerance of uncertainty that uses subscales to measure (1) anxiety due to uncertainty, (2) concern about bad outcomes, (3) reluctance to disclose uncertainty to patients, and (4) reluctance to disclose mistakes to physicians (eTable 1 in Supplement 1).^18,21^ As physician anxiety due to uncertainty was the construct among the PRU scales we conceptualized as most related to consultation use, we specifically evaluated responses to this construct, consistent with a prior study of uncertainty tolerance among pediatric hospitalists.^22^ The anxiety due to uncertainty scale ranges from 6 to 30, with higher scores indicating greater anxiety.

#### EHR Review

##### Patient Hospitalizations

We evaluated hospitalizations to general pediatric inpatient services between October 1, 2015, and December 31, 2020. For hospitalizations longer than 10 days, we limited analysis to the first 10 hospital days, by which time subspecialty services would have been expected to consult for the primary presenting concern in a typical hospitalization. To allow for comparison of consultation use across patients and across physicians, we targeted well-defined cohorts of general pediatrics patients. Using identifiable *International Statistical Classification of Diseases and Related Health Problems, Tenth Revision *(*ICD-10*)–based criteria used in large studies of hospitalized children (eTable 2 in Supplement 1),^23,24,25,26,27,28,29^ we included hospitalizations for any of the following 15 common pediatric conditions: asthma, bronchiolitis, cervical lymphadenitis, constipation, croup, deep neck space infection, febrile infant, gastroenteritis, Kawasaki disease, orbital or preseptal cellulitis, osteomyelitis, pneumonia, septic arthritis, skin and soft-tissue infection (SSTI), and urinary tract infection (UTI). For the small proportion (2%) of hospitalizations that met definitions for 2 conditions, we applied consensus criteria (eTable 3 in Supplement 1) or, if criteria could not be applied, 2-physician review to assign the condition expected to primarily inform consultation practices. We excluded hospitalizations with intensive care unit (ICU) use, hospitalizations among patients with complex chronic conditions (CCCs),^30^ and 30-day hospital readmissions for the same condition as the index hospitalization.

##### Consultation Identification

New consultations were identified by the presence of an initial subspecialty consultation note by a previously uninvolved service. We excluded consultations performed by nonphysician services (eg, chaplain, occupational therapist) or without explicit request from hospitalist physicians to address a clinical question (eg, anesthesia consultations associated with procedures) (eTable 4 in Supplement 1).

##### Consultation Attribution

To attribute consultations, we first identified the attending of record listed in the EHR for each date and time. Given the potential for incorrect assignment of the attending of record in the EHR, we validated the EHR attending of record against the attending physicians who had finalized a general pediatrics clinical note during the hospitalization. Patient-days for which there was a mismatch between these sources (4% of total) were reconciled with our institution’s shift schedule and the patient medical record, as needed. Clinical schedules were unavailable between October 1, 2015, and March 30, 2016, and to avoid inaccurate attribution, patient-days with mismatches during this period were excluded from analysis. Patient-days not corresponding to physicians who participated in the survey were also excluded from analysis.

After identifying the attending of record, for each new consultation note we applied the following approach to consultation attribution (eFigure in Supplement 1):

1. Consultations with a note initiation time stamp occurring when the attending physician was an emergency medicine attending (cross-checked with EHR specialty data) were attributed as emergency department (ED) consultations.
2. For remaining consultation notes, we evaluated for a corresponding specialty consultation order during the 24 hours preceding the consultation note initiation time stamp. We attributed the consultation to the attending of record at the time of the consultation order. We took this approach because while a consultation note indicates the presence of a consultation, a consultation order better reflects the timing of when a consultation is requested.
3. In the absence of a consultation order, we attributed consultations to the attending of record at the initial consultation note time stamp if the physician was also the attending of record in the 24 hours prior to this time stamp. We rationalized that even in the absence of a preceding consultation order, this attending could be assumed to be responsible for the consultation. We reviewed each patient medical record to confirm accurate assignment of the consultation date.
4. For consultations that remained unattributed, we performed medical record review and attributed consultations based on evaluation of clinical documentation and clinician orders. A sample of 40 randomly selected consultations reviewed independently by 2 physician reviewers (A.K.-G. and E.D.) demonstrated concordant attending attribution in 39 cases (97.5% agreement).

#### Patient, Physician, Admission, and Systems Characteristics

We evaluated characteristics hypothesized to contribute to consultation (eTable 5 in Supplement 1) including patient age, race and ethnicity (Hispanic, non-Hispanic Black, non-Hispanic White, and non-Hispanic other [any race listed in the medical record besides Black or White, including Asian, American Indian or Alaska Native, or other]),^31,32,33^ sex, payer (Medicaid, private, or unknown), care team model (resident/APP or frontline hospitalist team), day of the week, and year. As the clinical significance of a patient’s age depends on the condition of interest, we identified whether the patient age was an outlier (≤5th percentile or ≥95th percentile) for the age distribution of their condition. As the likelihood of consultation may change based on prior consultations, we computed prior consultations (including ED consultations) during the admission.

Physician characteristics were extracted from the survey. We calculated physician experience at the time of a hospitalization based on the years of experience reported in the survey and the date of the hospitalization. Because we were most interested in a physician’s anxiety due to uncertainty relative to other physicians (rather than the raw score), we categorized anxiety scores by quartile among responding physicians.

### Statistical Analysis

#### Primary Analysis

Because we were unaware of established methods used to examine consultation practices, we assessed our data to optimize our analytic approach. First, we examined the distribution of consultations per patient-day. As the vast majority of patient-days (99.2%) had either 0 or 1 consultation, our primary analysis applied logistic regression to evaluate the likelihood of consultation on a given patient-day as a binary outcome (consultation vs no consultation). Second, we conceptualized patient-days as potentially being clustered by patient or by physician and evaluated within-patient and within-physician clustering of observations using intraclass correlation coefficients.^34,35^ After comparing the proportion of variability attributed to differences across patients vs differences across physicians, we adopted a model accounting for patient-level clustering (consistent with prior literature^36^). Thus, we included the prespecified patient-, physician-, admission-, and systems-level factors in bivariable and multivariable generalized linear mixed models (GLMMs) for logistic regression using a random intercept term for patient. Using the models, we computed the odds of 1 or more consultations on a given patient-day. Statistical significance was set at *P* < .05, and tests were 2-tailed. All analyses were conducted using Stata/IC version 16.0 (StataCorp).

#### Sensitivity Analyses

We performed sensitivity analyses to further explore findings. Given the complex relationship between race and ethnicity and insurance status, we evaluated for interactions between these variables and performed an analysis excluding insurance status. To examine the association of these variables with consultation use within the data for each patient condition, we also fit logistic regression models conditional on patient condition (without physician characteristics). Lastly, due to the unique epidemiology of respiratory illnesses and changes to clinical practice due to the COVID-19 pandemic in 2020, we performed a sensitivity analysis excluding this year.

#### Subgroup Analyses

We also performed 2 prespecified subgroup analyses. First, to identify whether factors associated with consultation differed within the subset of conditions in which consultations are more routine, we performed a subanalysis excluding conditions in which fewer than 10% of hospitalizations contained a consultation (ie, asthma, bronchiolitis, croup, and pneumonia). Second, to better delineate factors associated with consultation intensity on patient-days in which a physician requested 1 or more consultations, we used a GLMM for logistic regression to identify factors associated with of 1 vs 2 or more consultations in this subset of patient-days. Model selection followed the same procedures described in the primary analysis.

#### Physician Comparisons

We calculated consultation rates for each physician by computing the number of patient-days with at least 1 consultation for every 100 patient-days attributed to that physician. We choose 100 patient-days as the denominator for this rate to permit comparison between physicians for a typical 7-day week on service with an average of 14 to 15 patients each day. We compared consultation rates in a standardized way by fitting a GLMM for logistic regression with physician as a fixed effect and calculating the estimated probabilities for each physician as if all patients were treated by this physician.^36^ We also compared the highest and lowest quartile of raw and adjusted consultation use using 2-sample *t* tests. To permit a meaningful sample size for each physician, we limited comparisons to physicians with at least 100 patient-days.

## Results

**Figure 1.**
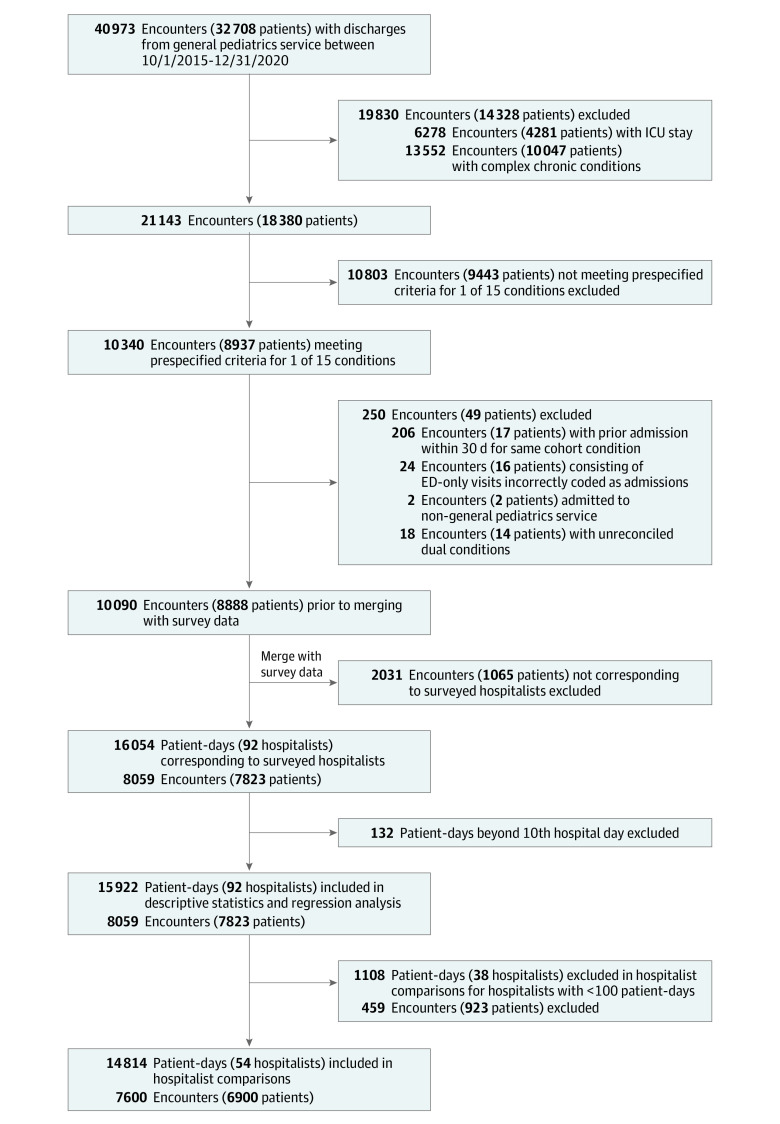
Study Flow Diagram ED indicates emergency department; ICU, intensive care unit.

Our cohort consisted of 15 922 patient-days across 8059 hospitalizations and 7283 unique patients (Figure 1); 3955 patients (54%) were male, 3450 (47%) were non-Hispanic Black and 2174 (30%) were non-Hispanic White, with a median (IQR) age of 2.5 (0.9-6.5) years (Table 1). Asthma (2770 patients [34%]) and bronchiolitis (1802 patients [22%]) accounted for more than half of admissions. Distribution of patient race and ethnicity and insurance status by condition is displayed in eTable 6 in Supplement 1.

**Table 1.** Patient, Admission, Physician, and Systems Characteristics

Variable	No. (%)^a^
Patient characteristics (n = 7283 patients)	
Sex	
Male	3955 (54)
Female	3328 (46)
Age, median (IQR), y	2.5 (0.9-6.5)
Race and ethnicity	
Hispanic	818 (11)
Non-Hispanic	
Black	3450 (47)
White	2174 (30)
Unknown	54 (<1)
Non-Hispanic other^b^	787 (11)
Admission characteristics (n = 8059 admissions)	
Condition	
Asthma	2770 (34)
Bronchiolitis	1802 (22)
Cervical lymphadenitis	205 (3)
Constipation	205 (3)
Croup	278 (3)
Deep neck space infection	114 (1)
Febrile infant	130 (2)
Gastroenteritis	229 (3)
Kawasaki disease	119 (1)
Orbital or preseptal cellulitis	212 (3)
Osteomyelitis	147 (2)
Pneumonia	904 (11)
Septic arthritis	47 (1)
SSTI	649 (8)
UTI	248 (3)
Insurance	
Medicaid	4936 (61)
Private Insurance	3000 (37)
Unknown	123 (2)
Year	
2015-2016^c^	2105 (26)
2017	1568 (19)
2018	1684 (21)
2019	1757 (22)
2020	945 (12)
Inpatient LOS, median (IQR), d	2 (2-3)
Physician characteristics (n = 92 physicians)	
Attending experience, y	
0-2	18 (20)
3-10	41 (45)
11-20	27 (29)
21-40	6 (7)
Anxiety due to uncertainty, quartile	
First (bottom; score range 6-13)	27 (29)
Second (score range 14-17)	20 (22)
Third (score range 18-20)	27 (29)
Fourth (top; score range 21-27)	18 (20)
Gender	
Male	23 (25)
Female	68 (74)
Prefer not to say	1 (1)
Systems characteristics (n = 15 922 patient-days)	
Inpatient hospital day	
1	3392 (21)
2-3	9890 (62)
4-6	2299 (14)
7-10	341 (2)
Day of the week	
Monday or Tuesday	7085 (44)
Wednesday, Thursday, or Friday	4508 (28)
Saturday or Sunday	4329 (27)
Team category	
Resident/APP	10 637 (67)
Frontline hospitalist	5285 (33)
No. of prior consultations during admission	
0	12 155 (76)
1	2283 (14)
≥2	1484 (9)

Abbreviations: APP, advanced practice practitioner; LOS, length of stay; SSTI, skin and soft-tissue infection; UTI, urinary tract infection.

^a^ Percentages may not equal 100% due to rounding.

^b^ Includes any race listed in the medical record other than White or Black (Asian, American Indian or Alaska Native, or other).

^c^ Years combined in analysis, as start of study period was October 1, 2015.

Among pediatric hospitalist physicians, 95 of 102 participated in the survey (93% response rate). Three surveyed physicians were not attributed to patient-days in the data set (eg, new attendings and/or exclusively caring for patients on complex care services who met exclusion criteria), leaving 92 physicians represented in the final data set. There were 68 (74%) women, and most physicians (74 [80%]) had 3 or more years of attending experience. Physician anxiety due to uncertainty scores ranged from 6-13 (first quartile) to 21-27 (fourth quartile). Teams with resident physicians were responsible for 10 637 patient-days (67%).

Of the 15 922 patient-days, 14 809 (93%) were associated with 0 consultations, 958 (6%) with 1 consultation, and 155 (1%) with 2 or more consultations. Frequency of consultation by condition is described in eTable 7 in Supplement 1. In the adjusted multivariable analysis, physician quartile of anxiety due to uncertainty was not significantly associated with consultation. However, we observed higher odds or consultation among physicians with 0 to 2 years of attending physician experience vs 3 to 10 years (odds ratio [OR], 1.42 [95% CI, 1.08-1.88]; *P* = .01), among male patients (OR, 1.22 [95% CI, 1.04-1.43]; *P* = .01), among patients with private insurance (OR, 1.19 [95% CI, 1.01-1.42]; *P* = .04), among those receiving care on a resident/APP team (OR, 1.27 [95% CI, 1.06-1.52]; *P* = .01), and those receiving care in later years (eg, 2020 vs 2015-2016: OR, 1.44 [95% CI, 1.11-1.87]; *P* = .006) (Table 2). Weekend care vs Monday or Tuesday care had lower odds of consultation (OR, 0.78 [95% CI, 0.65-0.94]; *P* = .008).

**Table 2.** Consultation Use Among Pediatric Hospitalists by Patient, Admission, Physician, and Systems Characteristics

Variable	No. of patient-days with consultation, No./total No. (%)^a^	Unadjusted				Adjusted			
		OR (95% CI)	*P* value			Adjusted OR (95% CI)	*P* value		
			Category		Composite		Category		Composite
**Patient characteristics**									
Patient sex									
Male	603/8562 (7)	1.02 (0.90-1.16)	.76	.76		1.22 (1.04-1.43)	.01	.01	
Female	510/7360 (7)	1 [Reference]	NA			1 [Reference]	NA		
Age outlier for condition^b^									
Yes	131/1616 (8)	1.20 (0.98-1.47)	.08	.08		1.26 (0.99-1.61)	.06	.06	
No	982/14 306 (7)	1 [Reference]	NA			1 [Reference]	NA		
Race and ethnicity									
Non-Hispanic									
White	505/5011 (10)	2.45 (2.10-2.85)	<.001	<.001		1.13 (0.93-1.38)	.22	.64	
Black	327/7373 (4)	1 [Reference]	NA			1 [Reference]	NA		
Other	153/1692 (9)	2.18 (1.77-2.69)	<.001			1.11 (0.86-1.44)	.42		
Hispanic	120/1736 (7)	1.60 (1.28-2.01)	<.001			0.96 (0.73-1.26)	.76		
Unknown	8/110 (7)	1.74 (0.81-3.72)	.15			0.89 (0.37-2.15)	.79		
**Admission characteristics** ^c^									
Insurance									
Medicaid	481/9469 (5)	1 [Reference]	NA	<.001		1 [Reference]	NA	.06	
Private	611/6224 (10)	2.05 (1.80-2.33)	<.001			1.19 (1.01-1.42)	.04		
Unknown	21/229 (9)	1.90 (1.17-3.08)	.009			1.54 (0.86-2.74)	.14		
Year									
2015-2016^d^	226/4079 (6)	1 [Reference]	NA	<.001		1 [Reference]	NA	.01	
2017	224/3077 (7)	1.34 (1.10-1.64)	.005			1.05 (0.83-1.33)	.70		
2018	246/3335 (7)	1.36 (1.12-1.66)	.002			1.27 (1.00-1.60)	.047		
2019	234/3484 (7)	1.22 (1.00-1.49)	.05			0.99 (0.78-1.25)	.91		
2020	183/1947 (9)	1.77 (1.43-2.20)	<.001			1.44 (1.11-1.87)	.006		
**Physician characteristics**									
Attending experience, y									
0-2	114/1362 (8)	1.30 (1.04-1.63)	.02	.12		1.42 (1.08-1.88)	.01	.05	
3-10	494/7444 (7)	1 [Reference]	NA			1 [Reference]	NA		
11-20	403/5768 (7)	1.05 (0.91-1.21)	.52			1.00 (0.84-1.18)	.96		
21-40	102/1348 (8)	1.14 (0.90-1.44)	.28			1.19 (0.90-1.59)	.23		
Anxiety due to uncertainty, quartile									
First, bottom	446/6255 (7)	1 [Reference]	NA	.89		1 [Reference]	NA	.59	
Second	199/2840 (7)	0.99 (0.82-1.19)	.90			0.91 (0.73-1.14)	.42		
Third	303/4328 (7)	0.99 (0.84-1.16)	.85			1.00 (0.83-1.21)	.98		
Fourth, top	165/2499 (7)	0.92 (0.76-1.12)	.43			0.86 (0.68-1.10)	.24		
Gender									
Male	319/4679 (7)	0.97 (0.84-1.12)	.71	.38		0.93 (0.78-1.12)	.45	.24	
Female	772/11 000 (7)	1 [Reference]	NA			1 [Reference]	NA		
Prefer not to say	22/243 (9)	1.37 (0.85-2.20)	.20			1.54 (0.84-2.80)	.16		
**Systems characteristics**									
Inpatient hospital day									
1	431/3392 (13)	1 [Reference]	NA	<.001		1 [Reference]	NA	<.001	
2-3	565/9890 (6)	0.36 (0.31-0.42)	<.001			0.35 (0.29-0.41)	<.001		
4-6	98/2299 (4)	0.19 (0.15-0.25)	<.001			0.17 (0.13-0.23)	<.001		
7-10	19/341 (6)	0.19 (0.11-0.33)	<.001			0.24 (0.14-0.42)	<.001		
Day of the week									
Monday or Tuesday	476/7085 (7)	1 [Reference]	NA	.005		1 [Reference]	NA	<.001	
Wednesday, Thursday, or Friday	361/4508 (8)	1.21 (1.04-1.40)	.01			1.13 (0.95-1.34)	.17		
Saturday or Sunday	276/4329 (6)	0.93 (0.79-1.09)	.36			0.78 (0.65-0.94)	.008		
Team category									
Resident/APP	778/10 637 (7)	1.17 (1.01-1.34)	.03	.03		1.27 (1.06-1.52)	.01	.01	
Frontline hospitalist	335/5285 (6)	1 [Reference]	NA			1 [Reference]	NA		
No. of prior consultations during admission									
0	696/12 155 (6)	1 [Reference]	NA	<.001		1 [Reference]	NA	<.001	
1	298/2283 (13)	2.45 (2.10-2.85)	<.001			0.46 (0.36-0.58)	<.001		
≥2	119/1484 (8)	1.37 (1.03-1.83)	.03			0.11 (0.07-0.17)	<.001		

Abbreviations: APP, advanced practice practitioner; NA, not applicable; OR, odds ratio.

^a^ Overall, 1113 of 15 922 patient-days (7%) had consultations.

^b^ Age in 5th percentile or less or 95th percentile or greater for condition.

^c^ Consultation use by patient condition displayed separately in the eAppendix in Supplement 1.

^d^ Years combined in analysis as start of study period was October 1, 2015.

In sensitivity analyses, race and ethnicity and insurance status did not have significant interaction effects. In our model excluding insurance status, non-Hispanic White patients had greater odds of consultation compared with non-Hispanic Black patients (OR, 1.23 [95% CI, 1.02-1.47]; *P* = .03) (eTable 8 in Supplement 1). Models conditional on patient condition, including and excluding insurance status, demonstrated relative stability of OR point estimates (eTables 9 and 10 in Supplement 1). Our model excluding 2020 did not reveal additional factors associated with consultation (eTable 11 in Supplement 1).

The subanalysis excluding low-consulting conditions (pneumonia, croup, bronchiolitis, and asthma) demonstrated similar findings as the primary analysis (eTable 12 in Supplement 1). In the subanalysis of patient-days with at least 1 consultation (eTable 13 in Supplement 1), non-Hispanic White patients (adjusted OR, 2.23 [95% CI, 1.23-4.13]; *P* = .01) and non-Hispanic patients of other race and ethnicity (adjusted OR, 2.56 [95% CI, 1.19-5.53]; *P* = .02) had greater odds of receiving multiple consultations compared with non-Hispanic Black patients.

**Figure 2.**
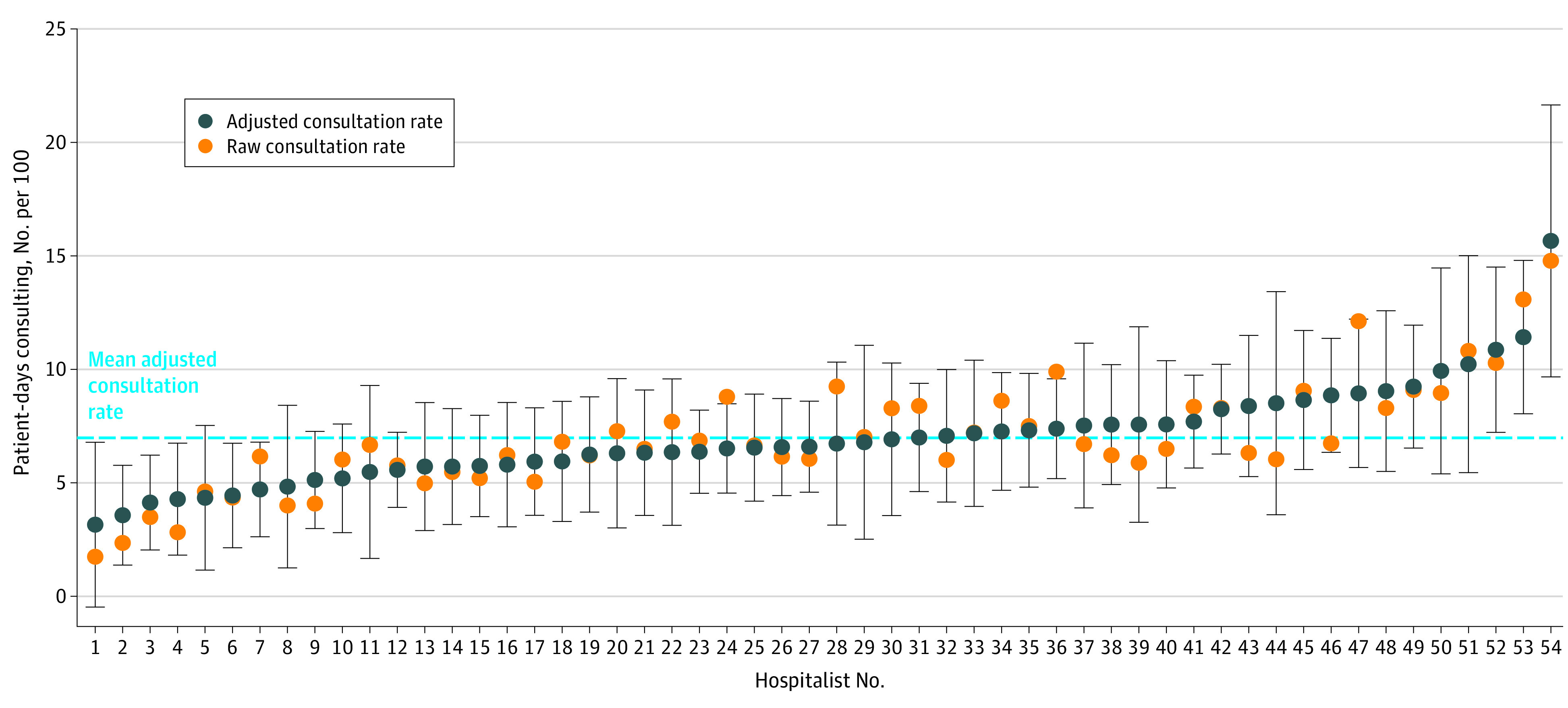
Comparison of Consultation Use Among Pediatric Hospitalist Physicians Data points represent the raw (orange) and adjusted (blue) consultation rates corresponding to each pediatric hospitalist physician, with vertical bars depicting the 95% CI for the adjusted consultation rate. Consultation rates represent the number of patient-days consulting (with ≥1 consult) for every 100 patient-days attributed to that physician. Physicians are ordered by adjusted consultation rate. The dashed blue line represents the mean adjusted consultation rate across all physicians.

In our comparison of hospitalist physicians, physicians in the top quartile of consultation use (mean [SD], 10.2 [2.0] patient-days consulting per 100) had consultation rates 2.3 times higher than physicians in the bottom quartile of consultation use (mean [SD], 4.3 [1.3] patient-days consulting per 100; *P* < .001). After adjustment for covariates, physicians in the top quartile of consultation use (mean [SD], 9.8 [2.0] patient-days consulting per 100) had consultation rates 2.1 times higher than physicians in the bottom quartile of consultation use (mean [SD], 4.7 [0.8] of 100 patient-days; *P* < .001). Figure 2 illustrates the distribution of adjusted consultation rates and superimposed raw consultation rates corresponding to each physician.

## Discussion

In this single-center study combining a cross-sectional survey of pediatric hospitalist physicians with retrospective EHR-derived data, we found that physician consultation varied more than 2-fold between the highest and lowest quartiles of consultation use. Contrary to our hypothesis, physician anxiety due to uncertainty was not associated with consultation use.

We identified patient, physician, admission, and systems factors associated with differential consultation use. At the patient level, while higher consultation use observed for male patients may reflect actual or perceived differences in illness presentation or severity,^37,38^ we cannot exclude residual confounding. Meanwhile, the higher consultation use identified among patients with private insurance vs Medicaid (also observed by Kachman et al among adult patients^10^) appears consistent with historically decreased utilization of health care resources among hospitalized Medicaid patients.^39,40^ At the physician level, we observed higher consultation use among physicians with the least attending experience (0-2 years), suggesting the need for focused study of how newer hospitalist physicians use consultations and the outcomes of proposed initiatives to enhance support for early-career physicians, such as mentoring or coaching programs,^41^ on enhancing value in consultation. At the admission level, our study demonstrated a marked increase in consultation use in 2020, even after adjustment for mix of conditions, which may correspond to increased subspecialist accessibility due the temporary use of remote consultation for some services during the height of the COVID-19 pandemic at our institutions, increased subspecialty billing to compensate for decreased patient volumes, and/or disproportionate admission of higher-acuity patients at this time.

We did not observe racial and ethnic differences in consultation use in the primary analysis but found higher odds of consultation among non-Hispanic White patients compared with non-Hispanic Black patients in our sensitivity analysis excluding insurance status. We also observed higher odds of multiple consultations among patients of non-Hispanic White and non-Hispanic other race and ethnicity compared with non-Hispanic Black patients in the subanalysis of patient-days with at least 1 consultation, which may suggest that when hospitalists seek subspecialty involvement for non-Hispanic Black patients, there may be lower intensity of consultation. Together, our findings suggest that consultation patterns may be influenced by the complex interplay between the intersection of anti-Black racism, patient trust and engagement in health care, language barriers, and differential consultation advocacy for families with higher vs lower resources. Urgent study is needed to further investigate how these factors may affect consultation patterns.

The lack of overall significant association between physician anxiety due to uncertainty with consultation use suggests that established associations between diagnostic uncertainty and test utilization^42,43,44^ might not extrapolate to inpatient consultation in a straightforward manner. The role of uncertainty in consultation is, no doubt, complex. For example, a physician tolerant of uncertainty may be more likely to consultationsubspecialists to expand working differential diagnoses (thereby generating more uncertainty), rather than converge on a working diagnosis earlier without subspecialty input. Moreover, while the PRU is a validated instrument and tolerance to uncertainty has historically been thought of as a trait-level characteristic (as opposed to a temporary state) of individuals,^45^ there is some evidence that it can change over time among physicians,^46^ which in this study would be expected to bias any associations with consultation use toward the null. Additionally, our study did not measure uncertainty at the patient level but rather a physician’s general psychology.

A strength of this study is analysis of consultation use at the patient-day level, a novel approach that builds upon prior work by Kachman et al^10^ using EHR data, which demonstrated variability in the admission-level frequency of consultations on general adult medicine services. By using EHR attending-of-record data rather than the author of inpatient admission notes, we believe our approach allowed for more accurate consultation attribution, including differentiation between hospitalist-initiated and ED-initiated consultations and between multiple hospitalists caring for the same patient during the course of a hospitalization. Our approach also permitted characterizing variability in consultation use among individual hospitalist physicians.

### Limitations

There are some limitations of this study. First, we defined patient conditions using *ICD-10* discharge diagnoses. While this approach allows for generalizability, it may not fully capture consultation practices among patients who have a suspected diagnosis of a condition but are ultimately not diagnosed with that condition. Second, there may be unmeasured confounding by indication that contributed to observed differences across patients and/or physicians. However, we restricted our sample to children without CCCs or ICU stays, adjusted for several factors related to case-mix, and accounted for unmeasured patient-specific factors within and between hospitalizations using mixed-effects modeling. Additionally, the modest difference observed between the raw and risk-adjusted variation in physician consultation use is consistent with the expected pseudorandom assignment of patients to physicians, which leads to similar patient distributions among physicians.^9^ Third, the aforementioned restriction of our sample as well as conduct of the study at a single-center limit generalizability to medically complex children and patients at other children’s hospitals where consultation practices may differ. Additionally, while we measured variation of consultation patterns as a potential indicator that unnecessary consultation was occurring, we did not directly measure how the outcomes of patients differed among highly consulted vs minimally consulted patients, nor do we understand the true value of a consultation in a patient and family-centered care model^47^ in which a risk of overuse must be balanced with the desire for an expert opinion. These represent crucial next steps.

## Conclusions

In this cohort study of hospitalized patients at a large children’s hospital, consultation use was highly variable and may not be attributable to patient need alone. Our detailed model of consultation offers specific targets for future efforts to increase value and equity in pediatric inpatient consultation. Future directions include obtaining multistakeholder perspectives on inpatient consultation practices, development of consultation best practice guidelines, and associating pediatric consultation practices with patient outcomes.

## Notes

Supplement 1. **eTable 1.** Physicians’ Reactions to Uncertainty (PRU) Scales **eTable 2.** Definitions of Conditions Included in Study Cohort **eTable 3. **Consensus Criteria to Select Condition for Hospitalizations Meeting 2 Condition Definitions **eTable 4.** Subspecialty Services and Provider Types Excluded from Definition of Consultation **eTable 5.** Variables Included in Primary Analysis **eTable 6. **Distribution of Hospital Admissions by Patient Race and Ethnicity, Insurance Status and Condition **eTable 7. **Consultation Rate by Condition **eTable 8.** Consultation Use Among Pediatric Hospitalists by Patient, Physician, Admission and Systems Characteristics, Excluding Insurance Status **eTable 9.** Consultation Use Among Pediatric Hospitalists by Patient, Admission and Systems Characteristics, Conditional on Patient Condition **eTable 10.** Consultation Use Among Pediatric Hospitalists by Patient, Admission and Systems Characteristics, Conditional on Patient Condition and Excluding Insurance Status **eTable 11. **Consultation Use Among Pediatric Hospitalists by Patient, Physician, Admission and Systems Characteristics, Excluding Year 2020 **eTable 12.** Consultation Use Among Pediatric Hospitalists by Patient, Physician, Admission and Systems Characteristics, Excluding Low-Consulting Conditions (Asthma, Bronchiolitis, Croup, Pneumonia) **eTable 13.** Comparison of Single vs Multiple Consultation by Pediatric Hospitalists Among Patient-Days with ≥1 Consultation **eFigure. **Methodology for Identifying Consultation Timing and Attending Attribution

Supplement 2.
Data Sharing Statement
