# Supplementary material for: Factors Associated With Inpatient Subspecialty Consultation Patterns Among Pediatric Hospitalists
Source: JAMA Netw Open. 2023 Mar 13;6(3):e232648. doi: 10.1001/jamanetworkopen.2023.2648 (PMC10011930; doi:10.1001/jamanetworkopen.2023.2648)
Supplement: Supplement 1. — eTable 1. Physicians’ Reactions to Uncertainty (PRU) Scales eTable 2. Definitions of Conditions Included in Study Cohort eTable 3. Consensus Criteria to Select Condition for Hospitalizations Meeting 2 Condition Definitions eTable 4. Subspecialty Services and Provider Types Excluded from Definition of Consultation eTable 5. Variables Included in Primary Analysis eTable 6. Distribution of Hospital Admissions by Patient Race and Ethnicity, Insurance Status and Condition eTable 7. Consultation Rate by Condition eTable 8. Consultation Use Among Pediatric Hospitalists by Patient, Physician, Admission and Systems Characteristics, Excluding Insurance Status eTable 9. Consultation Use Among Pediatric Hospitalists by Patient, Admission and Systems Characteristics, Conditional on Patient Condition eTable 10. Consultation Use Among Pediatric Hospitalists by Patient, Admission and Systems Characteristics, Conditional on Patient Condition and Excluding Insurance Status eTable 11. Consultation Use Among Pediatric Hospitalists by Patient, Physician, Admission and Systems Characteristics, Excluding Year 2020 eTable 12. Consultation Use Among Pediatric Hospitalists by Patient, Physician, Admission and Systems Characteristics, Excluding Low-Consulting Conditions (Asthma, Bronchiolitis, Croup, Pneumonia) eTable 13. Comparison of Single vs Multiple Consultation by Pediatric Hospitalists Among Patient-Days with ≥1 Consultation eFigure. Methodology for Identifying Consultation Timing and Attending Attribution [file jamanetwopen-e232648-s001.pdf]

Supplemental Online Content

Kern-Goldberger AS, Dalton EM, Rasooly IR, et al. Factors associated with inpatient subspecialty consultation patterns among pediatric hospitalists. *JAMA Netw Open.* 2023;6(3):e232648. doi:10.1001/jamanetworkopen.2023.2648

eTable 1. Physicians' Reactions to Uncertainty (PRU) Scales

eTable 2. Definitions of Conditions Included in Study Cohort

eTable 3. Consensus Criteria to Select Condition for Hospitalizations Meeting 2 Condition Definitions

eTable 4. Subspecialty Services and Provider Types Excluded from Definition of Consultation

eTable 5. Variables Included in Primary Analysis

eTable 6. Distribution of Hospital Admissions by Patient Race and Ethnicity, Insurance Status and Condition

eTable 7. Consultation Rate by Condition

eTable 8. Consultation Use Among Pediatric Hospitalists by Patient, Physician, Admission and Systems Characteristics, Excluding Insurance Status

eTable 9. Consultation Use Among Pediatric Hospitalists by Patient, Admission and Systems Characteristics, Conditional on Patient Condition

eTable 10. Consultation Use Among Pediatric Hospitalists by Patient, Admission and Systems Characteristics, Conditional on Patient Condition and Excluding Insurance Status

eTable 11. Consultation Use Among Pediatric Hospitalists by Patient, Physician, Admission and Systems Characteristics, Excluding Year 2020

eTable 12. Consultation Use Among Pediatric Hospitalists by Patient, Physician, Admission and Systems Characteristics, Excluding Low-Consulting Conditions (Asthma, Bronchiolitis, Croup, Pneumonia)

eTable 13. Comparison of Single vs Multiple Consultation by Pediatric Hospitalists Among Patient-Days with ≥1 Consultation

eFigure. Methodology for Identifying Consultation Timing and Attending Attribution

This supplemental material has been provided by the authors to give readers additional information about their work.

© 2023 Kern-Goldberger AS et al. *JAMA Network Open.*

eTable 1. Physicians' Reactions to Uncertainty (PRU) Scales

**Anxiety Due to Uncertainty (5 items)**
I usually feel anxious when I am not sure of a diagnosis
I find the uncertainty involved in patient care disconcerting.
Uncertainty in patient care makes me uneasy.
I am quite comfortable with the uncertainty in patient care.*
The uncertainty of patient care often troubles me.
**Concern About Bad Outcomes (3 items)**
When I am uncertain of a diagnosis, I imagine all sorts of bad scenarios - patient dies, patient sues, etc....
I fear being held accountable for the limits of my knowledge.
I worry about malpractice when I do not know a patient's diagnosis.
**Reluctance to Disclose Uncertainty to Patients (5 items)**
When physicians are uncertain of a diagnosis, they should share this information with their patients.*
I always share my uncertainty with my patients.*
If I shared all of my uncertainties with my patients, they would lose confidence in me.
Sharing my uncertainty improves my relationship with my patients.*
I prefer patients not know when I am uncertain of what treatments to use.
**Reluctance to Disclose Mistakes to Physicians (2 items)**
I almost never tell other physicians about diagnoses I have missed.
I never tell other physicians about patient care mistakes I have made.

Items scored on 6-point Likert scale (from 1=strongly disagree to 6=strongly agree). Items marked with asterisk (*) are reverse-scored (i.e. from 1=strongly agree to 6=strongly disagree). Scales are scored by summing the scores of individual items.

© 2023 Kern-Goldberger AS et al. *JAMA Network Open.*

eTable 2. Definitions of Conditions Included in Study Cohort

Condition	Definition
Gastroenteritis[a]	1. Age > 2mo, <6yo **and**<br>2. **either** of the following:<br>a. primary ICD-10 discharge diagnosis code of A08.4 (viral intestinal infection, unspecified), A08.8 (other specified intestinal infections), or A08.0 (rotaviral enteritis) **or**<br>b. primary ICD-10 discharge diagnosis code of E86.0 (dehydration) and secondary discharge diagnosis of viral gastroenteritis or rotavirus (A08.4, A08.0, A08.8)
Urinary tract infection (UTI)[a,b]	1. Age 2 mo–18 y **and**<br>2. Primary ICD-10 discharge diagnosis code of:<br>a. N10 (acute pyelonephritis)<br>b. N15.1 (renal or perinephric abscess)<br>c. N13.6 (pyonephrosis)<br>d. N12 (tubulointerstitial nephritis, not specified as acute or chronic)<br>e. N11.9 (chronic tubulointerstitial nephritis, unspecified)<br>f. N16 (renal tubulointerstitial disorders in diseases classified elsewhere<br>g. N39.0 (UTI, site not specified),<br>h. **or** P39.3 (neonatal UTI)
Constipation[a]	1. Age 2–18 y and either of the following:<br>a. Primary ICD-10 discharge diagnosis code of:<br>• K59.0x (constipation)<br>• K56.4x (other impaction of intestine),<br>• **or** F98.1 (encopresis)<br><br>**or**<br><br>b. **Secondary** discharge diagnosis code of any of the above with a **primary** discharge diagnosis code of:<br>• R11.2 (nausea and vomiting<br>• R11.10 (vomiting)<br>• K56.60x (unspecified intestinal obstruction)<br>• R15.x (incontinence of feces)<br>• K56.69x (other specified intestinal obstruction),<br>• K58.x (irritable bowel syndrome)<br>• K59.39 (other megacolon)<br>• K59.2 (neurogenic bowel, not otherwise classified)<br>• **or** K59.9 (functional intestinal disorders, unspecified)
Febrile infant[a]	1. Age <2 months and<br>2. Any ICD-10 discharge or admission diagnosis code for fever:<br>a. R50 (fever and other physiologic disturbances of temperature regulation)<br>b. P81.x (other disturbances of temperature regulation of newborn)<br>c. R50.9 (fever, unspecified),<br>d. **or** R50.81 (fever presenting with conditions classified elsewhere)
Pneumonia[a,b]	1. Age 2 months–18 years **and**<br>2. Discharge diagnosis of pneumonia defined by the following:<br>a. **Primary** discharge diagnosis code of<br>• pneumonia (J12.x, J13, J14, J15.x, J16.x, J18.0, J18.1, J18.8, J18.9)<br>• empyema (J86.x)<br>• **or** pleurisy (R09.1, J90, J91.8)<br><br>**or**<br>b. **Any** discharge diagnosis code of

© 2023 Kern-Goldberger AS et al. *JAMA Network Open.*

	• pneumonia (J12.x, J13, J14, J15.x, J16.x, J18.0, J18.1, J18.8, J18.9)<br>• empyema (J86.x)<br>• **or** pleurisy (R09.1, J90, J91.8)<br><br>**and**<br><br>Pneumonia-related **symptom** as the **primary** ICD-10-CM discharge code:<br>• Fever (R50.81 or R50.9)<br>• Respiratory abnormality, unspecified (R06.9)<br>• Shortness of breath (R06.02)<br>• Tachypnea (R06.82)<br>• Wheezing (R06.2)<br>• Cough (R05)<br>• Hemoptysis (R04.2, R04.9, or R04.89)<br>• Abnormal sputum (R09.3)<br>• Chest pain (R07.1, R07.8x, R07.9)<br>• Precordial pain (R07.2)<br>• Painful respiration (R07.1, R07.81)<br><br><u>Exclusions</u>:<br>• Diagnosis of trauma (J95.1, J95.2, J95.3, J95.821, J95.822, S00-T88)<br>• Aspiration pneumonia (J68-J70)<br>• Diagnoses associated with pregnancy or delivery (O00-09A)
Skin and soft tissue infection (SSTI)[a,b]	1. Age 2 <u>months</u>–18 <u>years</u>; **and**<br>2. Discharge diagnosis in **any position** starting with the following:<br>a. K12.2 (cellulitis and abscess of mouth)<br>b. L02.x (cutaneous abscess, furuncle and carbuncle);<br>c. L03.0, L03.1, L03.211, L03.212, L03.22, L03.3, L03.8, L03.9 (cellulitis and acute lymphangitis)<br>d. L98.3 (eosinophilic cellulitis)<br>e. K61.x (abscess of anal and rectal region);<br>f. P39.0 (neonatal infective mastitis); and<br>g. N61.x (inflammatory disorders of breast)<br><br><u>Exclusions</u>: in any position<br>1. PICC line (procedure code 02HV33Z, 02H633Z, or 06H033Z) performed during same encounter<br>2. hereditary/immuno: DX in any position starting with D80.x, D81.0, D81.1, D81.2, D81.4, D81.6, D81.7, D81.89, D81.9, D82.x, D83.x, D84.x, D89.3, D89.4x, D89.81x, D89.82, D89.89, D89.9, D71, and D72.0<br>3. HIV: DX in any position starting with Z21, and B20<br>4. malignant: DX in any position between C00 and D49<br>5. dacryocystitis: DX in any position starting with P39.1, H04.30x, H04.32x, H00.03x, and H04.00x<br>6. lymphadenitis: DX in any position starting with D75.8, L04.x, and Q18.0<br>7. omphalitis: DX in any position starting with P38.x<br>8. periorbital: L03.213<br>9. orbital: DX in any position starting with H05.01x<br>10. impetigo: DX in any position starting with L01.x
Bronchiolitis[a]	1. Age 2mo-2yr<br>2. Primary discharge diagnosis code of J21.x; and<br>3. No secondary discharge diagnosis codes for pneumonia (J12.x, J13, J14, J15.x, J16.x, J18.0, J18.1, J18.8, J18.9) or asthma (J45.x, J44.x)
Asthma[a]	1. Age 2–18 yr

© 2023 Kern-Goldberger AS et al. *JAMA Network Open.*

	2. **Primary** discharge diagnosis code of J45.x (asthma) **or** J44.x (chronic obstructive pulmonary disease), **and**<br>3. **No secondary** discharge diagnosis codes for pneumonia (J12.x, J13, J14, J15.x, J16.x, J18.0, J18.1, J18.8, J18.9) **or** bronchiolitis (J21.x)
Croup[c]	1. Ages 6mo- 6yo, **and**<br>2. Primary or secondary discharge diagnosis representing viral croup<br>• J04.2 (acute laryngotracheitis)<br>• J05.0 (Acute obstructive laryngitis [croup]<br>• J04.30 (Supraglottitis, unspecified, without obstruction)<br>• J04.31 (Supraglottitis, unspecified, with obstruction)<br>• **Or** J06.0 (Acute laryngopharyngitis)<br><br><u>Exclusions</u>:<br>Primary or secondary discharge diagnosis code for:<br>1. Asthma (J45.x or J44.x)<br>2. Pneumonia (J12.x, J13, J14, J15.x, J16.x, J18.0, J18.1, J18.8, J18.9, J86.x, R09.1, J90, J91.8)<br>3. Congenital anomalies of larynx/trachea (Q31-Q32)<br>4. Trauma, Burns, Foreign body aspiration/ingestion (S00-T88)<br>5. Surgical diagnosis, including tracheostomy complications (J95, Z43.0, Z93.0)<br>6. Motor vehicle accidents (V00-V99)
Kawasaki disease[d,e]	1. Discharge diagnosis code for Kawasaki Disease (M30.3), **and**<br>2. Received intravenous immunoglobulin (IVIG) during hospitalization<br><br><u>Exclusions</u>:<br>1. Admission on/after 3/15/2020 (to avoid confounding with newly identified MIS-C during COVID-19 pandemic)<br><br>2. Any of the following cardiac diagnoses:<br>• Q20 (Congenital malformations of cardiac chambers and connections)<br>• Q21.3 (Tetralogy of Fallot)<br>• Q22 (Congenital malformations of pulmonary and tricuspid valves)<br>• Q23 (Congenital malformations of aortic and mitral valves)<br>• Q24 (Other congenital malformations of heart)<br>• Q25 (Congenital malformations of great arteries)<br>• Q26 (Congenital malformations of great veins)
Orbital/preseptal cellulitis[b]	1. Age 2 months to 18 years, **and**<br>2. Discharge diagnosis of periorbital/orbital infection defined by primary or secondary<br>code of any one of the following:<br>• H05.01 (cellulitis of orbit)<br>• H05.02 (osteomyelitis of orbit)<br>• L03.213 (periorbital cellulitis)
Cervical lymphadenitis[f]	1. Primary or secondary discharge diagnosis of one of the following:<br>a. Acute lymphadenitis (L04.0, L04.9)<br>b. Cellulitis or abscess, carbuncle or furuncle of neck (L02.11-12, L03.221-222)<br>c. Chronic or nonspecific lymphadenitis (I88.1, I88.9)<br>d. Neck swelling, mass or lump (R22.1)<br>e. Enlarged lymph nodes (R59.9)<br><br><u>Exclusions</u>:<br>1. Patients who did not receive antibiotics during the hospitalization<br>2. Patients with CPT codes for CT, ultrasound, or MRI of chest, abdomen, pelvis, or extremities

© 2023 Kern-Goldberger AS et al. *JAMA Network Open.*

	3. Patients with discharge diagnosis codes for:<br>a. Kawasaki disease (M30.3)<br>b. Retropharyngeal/parapharyngeal abscess (J39.0)<br>c. Mastoiditis (H70, H75.0)<br>d. Dental abscess (K04.6, K04.7)<br>e. Lymphoma / other oncological diagnosis (C81 – C89)
Osteomyelitis[b]	1. Age 2 months to 18 years AND<br>2. Diagnoses of osteomyelitis defined by primary or secondary code of any one of the following:<br>a. A54.43 (gonococcal osteomyelitis)<br>b. M46.2x (osteomyelitis of vertebra)<br>c. M86.0x (acute osteomyelitis)<br>d. M86.1x (other acute osteomyelitis)<br>e. M86.2x (subacute osteomyelitis)<br>f. M86.4x (chronic osteomyelitis with draining sinus)<br>g. M86.5x (other chronic hematogenous osteomyelitis)<br>h. M86.6x (other chronic osteomyelitis)<br>i. M86.8x (other osteomyelitis)<br>j. M86.9 (osteomyelitis, unspecified)<br><br>Exclusions:<br>a. M86.29 (acute osteomyelitis, multiple sites)<br>b. M86.49 (chronic osteomyelitis with draining sinus, multiple sites)<br>c. M86.59 (other chronic hematogenous osteomyelitis, multiple sites)<br>d. M86.69 (other chronic osteomyelitis, multiple sites)
Deep neck space infection[g]	1. Primary or secondary discharge diagnosis code for J39.0 (Retropharyngeal and parapharyngeal abscess), **and**<br>2. At least 1 antibiotic administered on the first or second hospital day
Septic arthritis[b]	1. Age 2 months to 18 years AND<br>2. Discharge diagnoses of septic arthritis defined by primary or secondary code of any one of the following:<br>• A54.52 (gonococcal arthritis)<br>• M00.0x (staphylococcal arthritis and polyarthritis)<br>• M00.1x (pneumococcal arthritis and polyarthritis)<br>• M00.2x (other streptococcal arthritis and polyarthritis)<br>• M00.8x (arthritis and polyarthritis due other bacteria)<br>• M00.9x (pyogenic arthritis, unspecified)

[a] Adapted from Tchou et al., 2019
[b] Adapted from Cotter et al., 2021
[c] Adapted from Tyler et al., 2019 using Centers for Medicare and Medicaid Services general equivalency mapping of ICD-9-CM codes to ICD-10-CM codes
[d] Adapted from Coon et al., 2018
[e] Adapted from Hester et al., 2019
[f] Adapted from Desai et al., 2020
[g] Adapted from Lipsett et al., 2021

© 2023 Kern-Goldberger AS et al. *JAMA Network Open.*

eTable 3. Consensus Criteria to Select Condition for Hospitalizations Meeting 2 Condition Definitions

Condition 1	Condition 2	Cohort Selected	Hospitalizations affected	Rationale
Cervical lymphadenitis	SSTI	Cervical lymphadenitis	72	Cervical lymphadenitis likely driving consultation behavior
Osteomyelitis	SSTI	Osteomyelitis	35	Osteomyelitis is likely driving consultation behavior
Septic Arthritis	SSTI	Septic Arthritis	4	Septic arthritis is likely driving consultation behavior
Orbital/Periorbital Cellulitis	Osteomyelitis	Orbital/Periorbital Cellulitis	7	Orbital cellulitis with bony involvement is managed by Ophthalmology at our institution. For this group, we would expect consultation patterns similar to a diagnosis of orbital cellulitis alone.
Osteomyelitis	Septic Arthritis	Septic Arthritis	15	Concern for septic arthritis will likely drive consultation behavior (e.g., need for Orthopedic drainage/washout)

Abbreviations: SSTI = Skin and Soft Tissue Infection
N=27 hospitalizations meeting definitions for 2 conditions outside of those listed above reconciled via independent chart review by 2 physician reviewers.

© 2023 Kern-Goldberger AS et al. *JAMA Network Open.*

eTable 4. Subspecialty Services and Provider Types Excluded from Definition of Consultation

**Subspecialty Services**
Adult Care
Audiology
Critical Care
Nutrition Support
Sedation Services
Social Work
Speech Pathology
Vascular Access
Wound Ostomy
Cardiac Critical Care
Anesthesia
**Subspecialty Provider Types**
Audiologist
Behavioral Analyst
Chaplain
Clinical Nurse Specialist
Occupational Therapist
Physical Therapist
Registered Nurse
Social Worker
Social Worker Intern
Speech Therapist

© 2023 Kern-Goldberger AS et al. *JAMA Network Open.*

eTable 5. Variables Included in Primary Analysis

Variable	Categories	Source
Patient Characteristics		
Sex	Male<br>Female	EHR
Outlier age for cohort condition<br>(≤5th percentile or ≥95th percentile	Yes<br>No	EHR
Race/ethnicity	Non-Hispanic White<br>Non-Hispanic Black<br>Non-Hispanic Other<br>Hispanic<br>Unknown	EHR
Admission Characteristics		
Condition	Asthma<br>Bronchiolitis<br>Cervical lymphadenitis<br>Constipation<br>Croup<br>Deep neck space infection<br>Febrile infant<br>Gastroenteritis<br>Kawasaki disease<br>Orbital/preseptal cellulitis<br>Osteomyelitis<br>Pneumonia<br>Septic arthritis<br>SSTI<br>UTI	EHR
Insurance	Medicaid<br>Private Insurance<br>Unknown	EHR
Year	2015-2016[a]<br>2017<br>2018<br>2019<br>2020	EHR
Physician Characteristics		
Attending experience (years)	0-2<br>3-10<br>11-20<br>21-40	Survey[b]
Anxiety due to uncertainty quartile	1st (bottom) quartile<br>2nd quartile<br>3rd quartile<br>4th (top) quartile	Survey
Gender	Male<br>Female<br>Prefer not to say	Survey
Systems Characteristics		
Inpatient hospital day	1<br>2-3<br>4-6<br>7-10	EHR
Day of the week	Mon/Tues<br>Wed/Thurs/Fri	EHR

© 2023 Kern-Goldberger AS et al. *JAMA Network Open.*

	Sat/Sun	
Team category	Resident/APP<br>Frontline Hospitalist	EHR
Number of prior consultations during admission	0<br>1<br>≥2	

Abbreviations: EHR = electronic health record; SSTI = skin and soft tissue infection; UTI = urinary tract infection; APP = advanced practice practitioner

[a] Years combined in analysis as start of study period was October 1, 2015

[b] Number of years adjusted based on patient's admission date relative to survey completion

eTable 6. Distribution of Hospital Admissions by Patient Race and Ethnicity, Insurance Status and Condition

Condition		Insurance Status			
		Medicaid	Private	Unknown	**Total**
	**Race/Ethnicity**[a]	Hospital Admissions, n (%)[b,c]			
**Overall**	Non-Hispanic White	619 (8)	1,628 (20)	25 (0)	**2,272 (28)**
	Non-Hispanic Black	3,174 (39)	760 (9)	69 (1)	**4,003 (50)**
	Non-Hispanic Other	434 (5)	391 (5)	10 (0)	**835 (10)**
	Hispanic	683 (8)	194 (2)	17 (0)	**894 (11)**
	Unknown	26 (0)	27 (0)	2 (0)	**55 (1)**
	**Total**	**4,936 (61)**	**3,000 (37)**	**123 (2)**	**8,059 (100)**
**Asthma**	Non-Hispanic White	94 (3)	216 (8)	7 (0)	**317 (11)**
	Non-Hispanic Black	1,543 (56)	400 (14)	43 (2)	**1,986 (72)**
	Non-Hispanic Other	145 (5)	82 (3)	2 (0)	**229 (8)**
	Hispanic	174 (6)	45 (2)	4 (0)	**223 (8)**
	Unknown	11 (0)	3 (0)	1 (0)	**15 (1)**
	**Total**	**1,967 (71)**	**746 (27)**	**57 (2)**	**2,770 (100)**
Bronchiolitis	Non-Hispanic White	158 (9)	345 (19)	3 (0)	**506 (28)**
	Non-Hispanic Black	802 (44)	101 (6)	2 (0)	**905 (50)**
	Non-Hispanic Other	97 (5)	53 (3)	1 (0)	**151 (8)**
	Hispanic	194 (11)	39 (2)	1 (0)	**234 (13)**
	Unknown	2 (0)	4 (0)	0 (0)	**6 (0)**
	**Total**	**1,253 (70)**	**542 (30)**	**7 (0)**	**1,802 (100)**
Orbital/ Periorbital Cellulitis	Non-Hispanic White	19 (9)	75 (35)	4 (2)	**98 (46)**
	Non-Hispanic Black	39 (18)	18 (8)	0 (0)	**57 (27)**
	Non-Hispanic Other	9 (4)	16 (8)	1 (0)	**26 (12)**
	Hispanic	18 (8)	11 (5)	0 (0)	**29 (14)**
	Unknown	1 (0)	1 (0)	0 (0)	**2 (1)**
	**Total**	**86 (41)**	**121 (57)**	**5 (2)**	**212 (100)**
Cervical Lymphadenitis	Non-Hispanic White	32 (16)	55 (27)	0 (0)	**87 (42)**
	Non-Hispanic Black	55 (27)	15 (7)	3 (1)	**73 (36)**
	Non-Hispanic Other	13 (6)	15 (7)	0 (0)	**28 (14)**
	Hispanic	12 (6)	4 (2)	0 (0)	**16 (8)**
	Unknown	1 (0)	0 (0)	0 (0)	**1 (0)**
	**Total**	**113 (55)**	**89 (43)**	**3 (1)**	**205 (100)**
Constipation	Non-Hispanic White	14 (7)	55 (27)	0 (0)	**69 (34)**
	Non-Hispanic Black	63 (31)	14 (7)	3 (1)	**80 (39)**
	Non-Hispanic Other	10 (5)	15 (7)	0 (0)	**25 (12)**
	Hispanic	20 (10)	9 (4)	0 (0)	**29 (14)**
	Unknown	1 (0)	1 (0)	0 (0)	**2 (1)**
	**Total**	**108 (53)**	**94 (46)**	**3 (1)**	**205 (100)**

Croup	Non-Hispanic White	25 (9)	108 (39)	0 (0)	**133 (48)**
	Non-Hispanic Black	41 (15)	8 (3)	3 (1)	**52 (19)**
	Non-Hispanic Other	18 (6)	27 (10)	1 (0)	**46 (17)**
	Hispanic	34 (12)	10 (4)	1 (0)	**45 (16)**
	Unknown	0 (0)	2 (1)	0 (0)	**2 (1)**
	**Total**	**118 (42)**	**155 (56)**	**5 (2)**	**278 (100)**
Deep Neck Space Infection	Non-Hispanic White	11 (10)	45 (39)	0 (0)	**56 (49)**
	Non-Hispanic Black	30 (26)	9 (8)	0 (0)	**39 (34)**
	Non-Hispanic Other	4 (4)	4 (4)	0 (0)	**8 (7)**
	Hispanic	8 (7)	1 (1)	2 (2)	**11 (10)**
	Unknown	0 (0)	0 (0)	0 (0)	**0 (0)**
	**Total**	**53 (46)**	**59 (52)**	**2 (2)**	**114 (100)**
Febrile Infant	Non-Hispanic White	13 (10)	36 (28)	0 (0)	**49 (38)**
	Non-Hispanic Black	33 (25)	11 (8)	0 (0)	**44 (34)**
	Non-Hispanic Other	5 (4)	13 (10)	0 (0)	**18 (14)**
	Hispanic	14 (11)	4 (3)	0 (0)	**18 (14)**
	Unknown	1 (1)	0 (0)	0 (0)	**1 (1)**
	**Total**	**66 (51)**	**64 (49)**	**0 (0)**	**130 (100)**
Gastroenteritis	Non-Hispanic White	17 (7)	63 (28)	0 (0)	**80 (35)**
	Non-Hispanic Black	75 (33)	15 (7)	1 (0)	**91 (40)**
	Non-Hispanic Other	14 (6)	16 (7)	0 (0)	**30 (13)**
	Hispanic	24 (10)	1 (0)	0 (0)	**25 (11)**
	Unknown	0 (0)	3 (1)	0 (0)	**3 (1)**
	**Total**	**130 (57)**	**98 (43)**	**1 (0)**	**229 (100)**
Kawasaki **Disease**	Non-Hispanic White	8 (7)	34 (29)	0 (0)	**42 (35)**
	Non-Hispanic Black	22 (18)	11 (9)	1 (1)	**34 (29)**
	Non-Hispanic Other	10 (8)	17 (14)	0 (0)	**27 (23)**
	Hispanic	7 (6)	8 (7)	0 (0)	**15 (13)**
	Unknown	0 (0)	1 (1)	0 (0)	**1 (1)**
	**Total**	**47 (40)**	**71 (60)**	**1 (1)**	**119 (100)**
Osteomyelitis	Non-Hispanic White	16 (11)	69 (47)	1 (1)	**86 (58)**
	Non-Hispanic Black	18 (12)	10 (7)	0 (0)	**28 (19)**
	Non-Hispanic Other	5 (3)	14 (10)	0 (0)	**19 (13)**
	Hispanic	9 (6)	3 (2)	0 (0)	**12 (8)**
	Unknown	1 (1)	1 (1)	0 (0)	**2 (1)**
	**Total**	**49 (33)**	**97 (66)**	**1 (1)**	**147 (100)**
Pneumonia	Non-Hispanic White	64 (7)	250 (28)	5 (1)	**319 (35)**
	Non-Hispanic Black	249 (28)	81 (9)	5 (1)	**335 (37)**
	Non-Hispanic Other	55 (6)	52 (6)	2 (0)	**109 (12)**

© 2023 Kern-Goldberger AS et al. *JAMA Network Open.*

	Hispanic	98 (11)	26 (3)	6 (1)	**130 (14)**
	Unknown	6 (1)	5 (1)	0 (0)	**11 (1)**
	**Total**	**472 (52)**	**414 (46)**	**18 (2)**	**904 (100)**
Septic Arthritis	Non-Hispanic White	8 (17)	23 (49)	0 (0)	**31 (66)**
	Non-Hispanic Black	4 (9)	1 (2)	1 (2)	**6 (13)**
	Non-Hispanic Other	1 (2)	2 (4)	0 (0)	**3 (6)**
	Hispanic	4 (9)	2 (4)	0 (0)	**6 (13)**
	Unknown	0 (0)	1 (2)	0 (0)	**1 (2)**
	**Total**	**17 (36)**	**29 (62)**	**1 (2)**	**47 (100)**
SSTI	Non-Hispanic White	102 (16)	180 (28)	4 (1)	**286 (44)**
	Non-Hispanic Black	153 (24)	58 (9)	4 (1)	**215 (33)**
	Non-Hispanic Other	33 (5)	49 (8)	2 (0)	**84 (13)**
	Hispanic	36 (6)	19 (3)	2 (0)	**57 (9)**
	Unknown	2 (0)	4 (1)	1 (0)	**7 (1)**
	**Total**	**326 (50)**	**310 (48)**	**13 (2)**	**649 (100)**
UTI	Non-Hispanic White	38 (15)	74 (30)	1 (0)	**113 (46)**
	Non-Hispanic Black	47 (19)	8 (3)	3 (1)	**58 (23)**
	Non-Hispanic Other	15 (6)	16 (6)	1 (0)	**32 (13)**
	Hispanic	31 (12)	12 (5)	1 (0)	**44 (18)**
	Unknown	0 (0)	1 (0)	0 (0)	**1 (0)**
	**Total**	**131 (53)**	**111 (45)**	**6 (2)**	**248 (100)**

Abbreviations: SSTI = skin and soft tissue infection; UTI = urinary tract infection
[a] Includes any race listed in the medical record other than White or Black (Asian, American Indian or Alaska Native, Indian, or Other)
[b] Percentage reflects relative frequency of each cell
[c] Percentages may not sum to 100% due to rounding

© 2023 Kern-Goldberger AS et al. *JAMA Network Open.*

eTable 7. Consultation Rate by Condition[a]

Condition	No. of patient-days with consultation, n/N (%)
Asthma	36/4,179 (0.9)
Bronchiolitis	33/3,543 (0.9)
Cervical lymphadenitis	71/586 (12)
Constipation	51/428 (12)
Croup	11/373 (3)
Deep neck space infection	39/355 (11)
Febrile infant	43/353 (12)
Gastroenteritis	9/424 (2)
Kawasaki disease	119/438 (27)
Orbital/preseptal cellulitis	149/620 (24)
Osteomyelitis	165/569 (29)
Pneumonia	85/1,936 (4)
Septic arthritis	43/189 (23)
SSTI	199/1,370 (15)
UTI	60/559 (11)

Abbreviations: SSTI = skin and soft tissue infection; UTI = urinary tract infection
[a] Category p<0.001 in unadjusted and adjusted analyses

eTable 8. Consultation Use Among Pediatric Hospitalists by Patient, Physician, Admission and Systems Characteristics, Excluding Insurance Status

		Unadjusted			Adjusted		
			P value			*P* value	
**Variable**	**No. of patient-days with consultation, n/N (%)**	**OR (95% CI)**	**Category**	**Composite**	**Adjusted OR (95% CI)**	**Category**	**Composite**
**Overall**	1,113/15,922 (7)	NA	NA	NA	NA	NA	NA
Patient Characteristics							
**Patient sex**				0.76			0.02
Male	603/8,562 (7)	1.02 (0.90-1.16)	0.76		1.22 (1.04-1.43)	0.02	
Female	510/7,360 (7)	1 [Ref]	NA		1 [Ref]	NA	
**Age outlier for condition[a]**				0.08			0.05
Yes	131/1,616 (8)	1.20 (0.98-1.47)	0.08		1.28 (1.00-1.63)	0.05	
No	982/14,306 (7)	1 [Ref]	NA		1 [Ref]	NA	
**Race/Ethnicity**				<0.001			0.16
Non-Hispanic White	505/5,011 (10)	2.45 (2.10-2.85)	<0.001		1.23 (1.02-1.47)	0.03	
Non-Hispanic Black	327/7,373 (4)	1 [Ref]	NA		1 [Ref]	NA	
Non-Hispanic Other	153/1,692 (9)	2.18 (1.77-2.69)	<0.001		1.17 (0.91-1.51)	0.22	
Hispanic	120/1,736 (7)	1.60 (1.28-2.01)	<0.001		0.96 (0.74-1.26)	0.79	
Unknown	8/110 (7)	1.74 (0.81-3.72)	0.15		0.94 (0.39-2.27)	0.94	
Admission Characteristics							
**Condition[b]**				<0.001			<0.001
**Year**				<0.001			0.01
2015-2016[c]	226/4,079 (6)	1 [Ref]	NA		1 [Ref]	NA	
2017	224/3,077 (7)	1.34 (1.10-1.64)	0.005		1.05 (0.82-1.33)	0.71	
2018	246/3,335 (7)	1.36 (1.12-1.66)	0.002		1.27 (1.00-1.60)	0.047	
2019	234/3,484 (7)	1.22 (1.00-1.49)	0.05		0.98 (0.78-1.24)	0.89	
2020	183/1,947 (9)	1.77 (1.43-2.20)	<0.001		1.44 (1.11-1.87)	0.006	
Physician Characteristics							
**Attending experience (years)**				0.12			0.05
0-2	114/1,362 (8)	1.30 (1.04-1.63)	0.02		1.43 (1.08-1.89)	0.01	
3-10	494/7,444 (7)	1 [Ref]	NA		1 [Ref]	NA	
11-20	403/5,768 (7)	1.05 (0.91-1.21)	0.52		1.00 (0.84-1.20)	0.96	
21-40	102/1,348 (8)	1.14 (0.90-1.44)	0.28		1.20 (0.90-1.60)	0.22	
**Anxiety due to uncertainty quartile**				0.89			0.60
1st (bottom) quartile	446/6,255 (7)	1 [Ref]	NA		1 [Ref]	NA	
2nd quartile	199/2,840 (7)	0.99 (0.82-1.19)	0.90		0.91 (0.73-1.14)	0.41	
3rd quartile	303/4,328 (7)	0.99 (0.84-1.16)	0.85		1.00 (0.83-1.21)	0.995	
4th (top) quartile	165/2,499 (7)	0.92 (0.76-1.12)	0.43		0.87 (0.68-1.11)	0.26	
**Gender**				0.38			0.22
Male	319/4,679 (7)	0.97 (0.84-1.12)	0.71		0.93 (0.77-1.11)	0.41	
Female	772/11,000 (7)	1 [Ref]	NA		1 [Ref]	NA	
Prefer not to say	22/243 (9)	1.37 (0.85-2.20)	0.20		1.55 (0.85-2.81)	0.16	
Systems Characteristics							
**Inpatient hospital day**				<0.001			<0.001
1	431/3,392 (13)	1 [Ref]	NA		1 [Ref]	NA	
2-3	565/9,890 (6)	0.36 (0.31-0.42)	<0.001		0.35 (0.29-0.41)	<0.001	
4-6	98/2,299 (4)	0.19 (0.15-0.25)	<0.001		0.17 (0.13-0.23)	<0.001	

© 2023 Kern-Goldberger AS et al. *JAMA Network Open.*

7-10	19/341 (6)	0.19 (0.11-0.33)	<0.001		0.24 (0.14-0.42)	<0.001	
**Day of the week**				0.005			0.001
Mon/Tues	476/7,085 (7)	1 [Ref]	NA		1 [Ref]	NA	
Wed/Thurs/Fri	361/4,508 (8)	1.21 (1.04-1.40)	0.01		1.13 (0.95-1.34)	0.17	
Sat/Sun	276/4,329 (6)	0.93 (0.79-1.09)	0.36		0.78 (0.65-0.94)	0.008	
**Team category**				0.03			0.009
Resident/APP	778/10,637 (7)	1.17 (1.01-1.34)	0.03		1.27 (1.06-1.53)	0.009	
Frontline Hospitalist	335/5,285 (6)	1 [Ref]	NA		1 [Ref]	NA	
**Number of prior consultations during admission**				<0.001			<0.001
0	696/12,155 (6)	1 [Ref]	NA		1 [Ref]	NA	
1	298/2,283 (13)	2.45 (2.10-2.85)	<0.001		0.45 (0.36-0.58)	<0.001	
≥2	119/1,484 (8)	1.37 (1.03-1.83)	0.03		0.11 (0.07-0.17)	<0.001	

Abbreviations: ref, Reference; APP, advanced practice practitioner; NA, not applicable

[a] ≤5th percentile or ≥95th percentile age for condition

[b] Consultation use by patient condition displayed separately in Appendix

[c] Years combined in analysis as start of study period was October 1, 2015

© 2023 Kern-Goldberger AS et al. *JAMA Network Open.*

eTable 9. Consultation Use Among Pediatric Hospitalists by Patient, Admission and Systems Characteristics, Conditional on Patient Condition

		Unadjusted[a]			Adjusted		
			P value			*P* value	
**Variable**	**No. of patient-days with consultation, n/N (%)**	**OR (95% CI)**	**Category**	**Composite**	**Adjusted OR (95% CI)**	**Category**	**Composite**
**Overall**	1,113/15,922 (7)	NA	NA	NA	NA	NA	NA
Patient Characteristics							
**Patient sex**				0.09			0.02
Male	603/8,562 (7)	1.12 (0.98-1.28)	0.09		1.19 (1.03-1.37)	0.02	
Female	510/7,360 (7)	1 [Ref]	NA		1 [Ref]	NA	
**Age outlier for condition[b]**				0.05			0.04
Yes	131/1,616 (8)	1.22 (1.00-1.50)	0.05		1.26 (1.01-1.57)	0.04	
No	982/14,306 (7)	1 [Ref]	NA		1 [Ref]	NA	
**Race/Ethnicity**				0.59			0.69
Non-Hispanic White	505/5,011 (10)	1.11 (0.95-1.30)	0.18		1.12 (0.93-1.34)	0.23	
Non-Hispanic Black	327/7,373 (4)	1 [Ref]	NA		1 [Ref]	NA	
Non-Hispanic Other	153/1,692 (9)	1.14 (0.92-1.41)	0.23		1.08 (0.86-1.37)	0.50	
Hispanic	120/1,736 (7)	1.02 (0.81-1.28)	0.86		0.98 (0.76-1.25)	0.84	
Unknown	8/110 (7)	0.88 (0.41-1.88)	0.74		0.85 (0.37-1.93)	0.70	
Admission Characteristics							
**Insurance**				0.007			0.05
Medicaid	481/9,469 (5)	1 [Ref]	NA		1 [Ref]	NA	
Private	611/6,224 (10)	1.22 (1.06-1.39)	0.004		1.19 (1.02-1.39)	0.03	
Unknown	21/229 (9)	1.51 (0.93-2.45)	0.10		1.49 (0.88-2.52)	0.14	
**Year**				0.01			0.007
2015-2016[c]	226/4,079 (6)	1 [Ref]	NA		1 [Ref]	NA	
2017	224/3,077 (7)	1.16 (0.94-1.42)	0.16		1.07 (0.86-1.33)	0.53	
2018	246/3,335 (7)	1.33 (1.09-1.63)	0.005		1.26 (1.02-1.56)	0.03	
2019	234/3,484 (7)	1.10 (0.90-1.34)	0.37		1.01 (0.82-1.25)	0.93	
2020	183/1,947 (9)	1.38 (1.11-1.71)	0.004		1.43 (1.14-1.80)	0.002	
Systems Characteristics							
**Inpatient hospital day**				<0.001			<0.001
1	431/3,392 (13)	1 [Ref]	NA		1 [Ref]	NA	
2-3	565/9,890 (6)	0.26 (0.22-0.30)	<0.001		0.35 (0.30-0.42)	<0.001	
4-6	98/2,299 (4)	0.08 (0.06-0.10)	<0.001		0.17 (0.13-0.23)	<0.001	
7-10	19/341 (6)	0.09 (0.05-1.14)	<0.001		0.24 (0.14-0.41)	<0.001	
**Day of the week**				0.001			0.001
Mon/Tues	476/7,085 (7)	1 [Ref]	NA		1 [Ref]	NA	
Wed/Thurs/Fri	361/4,508 (8)	1.13 (0.97-1.32)	0.11		1.11 (0.95-1.31)	0.20	
Sat/Sun	276/4,329 (6)	0.83 (0.70-0.97)	0.02		0.79 (0.66-0.94)	0.008	
**Team category**				0.006			0.01
Resident/APP	778/10,637 (7)	1.22 (1.06-1.40)	0.006		1.21 (1.04-1.41)	0.01	
Frontline Hospitalist	335/5,285 (6)	1 [Ref]	NA		1 [Ref]	NA	
**Number of prior consultations during admission**							
0	696/12,155 (6)	1 [Ref]	NA	<0.001	1 [Ref]	NA	<0.001
1	298/2,283 (13)	0.34 (0.29-0.41)	<0.001		0.52 (0.43-0.63)	<0.001	
≥2	119/1,484 (8)	0.07 (0.06-0.10)	<0.001		0.16 (0.12-0.22)	<0.001	

Abbreviations: ref, Reference; APP, advanced practice practitioner; NA, not applicable

[a] Bivariable analyses also performed conditional on patient condition

[b] ≤5th percentile or ≥95th percentile age for condition

[c] Years combined in analysis as start of study period was October 1, 2015

© 2023 Kern-Goldberger AS et al. *JAMA Network Open.*

eTable 10. Consultation Use Among Pediatric Hospitalists by Patient, Admission and Systems Characteristics, Conditional on Patient Condition and Excluding Insurance Status

		Unadjusted[a]			Adjusted		
			P value			*P* value	
**Variable**	**No. of patient-days with consultation, n/N (%)**	**OR (95% CI)**	**Category**	**Composite**	**Adjusted OR (95% CI)**	**Category**	**Composite**
**Overall**	1,113/15,922 (7)	NA	NA	NA	NA	NA	NA
Patient Characteristics							
**Patient sex**				0.09			0.02
Male	603/8,562 (7)	1.12 (0.98-1.28)	0.09		1.19 (1.03-1.37)	0.02	
Female	510/7,360 (7)	1 [Ref]	NA		1 [Ref]	NA	
**Age outlier for condition[b]**				0.05			0.03
Yes	131/1,616 (8)	1.22 (1.00-1.50)	0.05		1.27 (1.02-1.58)	0.03	
No	982/14,306 (7)	1 [Ref]	NA		1 [Ref]	NA	
**Race/Ethnicity**				0.59			0.17
Non-Hispanic White	505/5,011 (10)	1.11 (0.95-1.30)	0.18		1.20 (1.02-1.42)	0.03	
Non-Hispanic Black	327/7,373 (4)	1 [Ref]	NA		1 [Ref]	NA	
Non-Hispanic Other	153/1,692 (9)	1.14 (0.92-1.41)	0.23		1.14 (0.90-1.43)	0.27	
Hispanic	120/1,736 (7)	1.02 (0.81-1.28)	0.86		0.98 (0.77-1.25)	0.87	
Unknown	8/110 (7)	0.88 (0.41-1.88)	0.74		0.90 (0.40-2.02)	0.79	
Admission Characteristics							
**Year**				0.01			0.006
2015-2016[c]	226/4,079 (6)	1 [Ref]	NA		1 [Ref]	NA	
2017	224/3,077 (7)	1.16 (0.94-1.42)	0.16		1.07 (0.86-1.33)	0.54	
2018	246/3,335 (7)	1.33 (1.09-1.63)	0.005		1.26 (1.02-1.56)	0.03	
2019	234/3,484 (7)	1.10 (0.90-1.34)	0.37		1.01 (0.81-1.24)	0.96	
2020	183/1,947 (9)	1.38 (1.11-1.71)	0.004		1.44 (1.14-1.80)	0.002	
Systems Characteristics							
**Inpatient hospital day**				<0.001			<0.001
1	431/3,392 (13)	1 [Ref]	NA		1 [Ref]	NA	
2-3	565/9,890 (6)	0.26 (0.22-0.30)	<0.001		0.35 (0.30-0.42)	<0.001	
4-6	98/2,299 (4)	0.08 (0.06-0.10)	<0.001		0.17 (0.13-0.23)	<0.001	
7-10	19/341 (6)	0.09 (0.05-1.14)	<0.001		0.24 (0.14-0.41)	<0.001	
**Day of the week**				0.001			0.001
Mon/Tues	476/7,085 (7)	1 [Ref]	NA		1 [Ref]	NA	
Wed/Thurs/Fri	361/4,508 (8)	1.13 (0.97-1.32)	0.11		1.11 (0.94-1.31)	0.21	
Sat/Sun	276/4,329 (6)	0.83 (0.70-0.97)	0.02		0.79 (0.67-0.94)	0.008	
**Team category**				0.006			0.01
Resident/APP	778/10,637 (7)	1.22 (1.06-1.40)	0.006		1.21 (1.04-1.41)	0.01	
Frontline Hospitalist	335/5,285 (6)	1 [Ref]	NA		1 [Ref]	NA	
**Number of prior consultations during admission**							
0	696/12,155 (6)	1 [Ref]	NA	<0.001	1 [Ref]	NA	<0.001
1	298/2,283 (13)	0.34 (0.29-0.41)	<0.001		0.52 (0.43-0.63)	<0.001	
≥2	119/1,484 (8)	0.07 (0.06-0.10)	<0.001		0.16 (0.12-0.22)	<0.001	

Abbreviations: ref, Reference; APP, advanced practice practitioner; NA, not applicable

[a] Bivariable analyses also performed conditional on patient condition

[b] ≤5th percentile or ≥95th percentile age for condition

[c] Years combined in analysis as start of study period was October 1, 2015

© 2023 Kern-Goldberger AS et al. *JAMA Network Open.*

eTable 11. Consultation Use Among Pediatric Hospitalists by Patient, Physician, Admission and Systems Characteristics, Excluding Year 2020

		Unadjusted			Adjusted		
			P value			*P* value	
**Variable**	**No. of patient-days with consultation, n/N (%)**	**OR (95% CI)**	**Category**	**Composite**	**Adjusted OR (95% CI)**	**Category**	**Composite**
**Overall**	930/13,975 (7)	NA	NA	NA	NA	NA	NA
Patient Characteristics							
**Patient sex**				0.71			0.02
Male	507/7,547 (7)	1.03 (0.89-1.19)	0.71		1.23 (1.03-1.46)	0.02	
Female	423/6,428 (7)	1 [Ref]	NA		1 [Ref]	NA	
**Age outlier for condition[a]**				0.22			0.17
Yes	104/1,385 (8)	1.16 (0.92-1.45)	0.22		1.20 (0.92-1.57)	0.17	
No	826/12,590 (7)	1 [Ref]	NA		1 [Ref]	NA	
**Race/Ethnicity**				<0.001			0.91
Non-Hispanic White	414/4,272 (10)	2.50 (2.11-2.96)	<0.001		1.08 (0.87-1.35)	0.48	
Non-Hispanic Black	274/6,568 (4)	1 [Ref]	NA		1 [Ref]	NA	
Non-Hispanic Other	137/1,543 (9)	2.29 (1.83-2.88)	<0.001		1.09 (0.82-1.43)	0.56	
Hispanic	97/1,491 (7)	1.60 (1.24-2.06)	<0.001		0.96 (0.72-1.29)	0.80	
Unknown	8/101 (8)	2.05 (0.94-4.44)	0.07		0.92 (0.38-2.26)	0.86	
Admission Characteristics							
**Condition[b]**		NA[b]		<0.001	NA[b]		
**Insurance**				<0.001			0.07
Medicaid	402/8,348 (5)	1 [Ref]	NA		1 [Ref]	NA	
Private	510/5,430 (9)	2.06 (1.79-2.38)	<0.001		1.18 (0.98-1.43)	0.08	
Unknown	18/197 (9)	2.01 (1.19-3.41)	0.009		1.73 (0.93-3.22)	0.08	
**Year**				0.01			0.13
2015-2016[c]	226/4,079 (6)	1 [Ref]	NA		1 [Ref]	NA	
2017	224/3,077 (7)	1.34 (1.09-1.65)	0.005		1.05 (0.82-1.33)	0.72	
2018	246/3,335 (7)	1.36 (1.11-1.66)	0.003		1.27 (1.01-1.61)	0.04	
2019	234/3,484 (7)	1.22 (1.00-1.50)	0.05		1.00 (0.79-1.26)	0.99	
Physician Characteristics							
**Attending experience (years)**				0.85			0.67
0-2	74/1,022 (7)	1.13 (0.86-1.49)	0.37		1.21 (0.87-1.68)	0.27	
3-10	437/6,697 (7)	1 [Ref]	NA		1 [Ref]	NA	
11-20	343/5,118 (7)	1.02 (0.87-1.19)	0.80		0.97 (0.81-1.17)	0.79	
21-40	76/1,138 (7)	1.01 (0.77-1.32)	0.93		1.04 (0.75-1.44)	0.81	
**Anxiety due to uncertainty quartile**				0.85			0.73
1st (bottom) quartile	382/5,616 (7)	1 [Ref]	NA		1 [Ref]	NA	
2nd quartile	153/2,364 (6)	0.95 (0.78-1.17)	0.64		0.93 (0.73-1.19)	0.58	
3rd quartile	260/3,828 (7)	1.00 (0.84-1.19)	0.98		1.01 (0.83-1.24)	0.91	
4th (top) quartile	135/2,167 (6)	0.92 (0.74-1.14)	0.44		0.88 (0.67-1.15)	0.34	
**Gender**				0.43			
Male	270/4,224 (6)	0.94 (0.81-1.10)	0.48		0.92 (0.76-1.12)	0.41	
Female	642/9,536 (7)	1 [Ref]	NA		1 [Ref]	NA	
Prefer not to say	18/215 (8)	1.32 (0.78-2.23)	0.31		1.37 (0.71-2.67)	0.35	
Systems Characteristics							
**Inpatient hospital day**				<0.001			<0.001

© 2023 Kern-Goldberger AS et al. *JAMA Network Open.*

1	369/2,999 (12)	1 [Ref]	NA		1 [Ref]	NA	
2-3	464/8,700 (5)	0.35 (0.29-0.41)	<0.001		0.32 (0.27-0.39)	<0.001	
4-6	81/1,988 (4)	0.19 (0.14-0.25)	<0.001		0.16 (0.12-0.22)	<0.001	
7-10	16/288 (6)	0.20 (0.11-0.36)	<0.001		0.25 (0.14-0.45)	<0.001	
**Day of the week**				0.01			0.004
Mon/Tues	397/6,264 (6)	1 [Ref]	NA		1 [Ref]	NA	
Wed/Thurs/Fri	302/3,939 (8)	1.22 (1.04-1.44)	0.01		1.19 (0.98-1.43)	0.07	
Sat/Sun	231/3,772 (6)	0.94 (0.79-1.12)	0.52		0.82 (0.67-1.01)	0.06	
**Team category**				0.16			0.03
Resident/APP	649/9,441 (7)	1.12 (0.96-1.31)	0.16		1.25 (1.03-1.52)	0.03	
Frontline Hospitalist	281/4,534 (6)	1 [Ref]	NA		1 [Ref]	NA	
**Number of prior consultations during admission**				<0.001			<0.001
0	590/10,801 (5)	1 [Ref]	NA		1 [Ref]	NA	
1	242/1,945 (12)	2.40 (2.03-2.85)	<0.001		0.44 (0.34-0.57)	<0.001	
≥2	98/1,229 (8)	1.35 (0.97-1.87)	0.07		0.12 (0.07-0.19)	<0.001	

Abbreviations: ref, Reference; APP, advanced practice practitioner; NA, not applicable

[a] ≤5th percentile or ≥95th percentile age for condition

[b] Consultation use by patient condition displayed separately in Appendix

[c] Years combined in analysis as start of study period was October 1, 2015

© 2023 Kern-Goldberger AS et al. *JAMA Network Open.*

eTable 12. Consultation Use Among Pediatric Hospitalists by Patient, Physician, Admission and Systems Characteristics, Excluding Low-Consulting Conditions (Asthma, Bronchiolitis, Croup, Pneumonia)

		Unadjusted			Adjusted		
			P value			*P* value	
**Variable**	**No. of patient-days with consultation, n/N (%)**	**OR (95% CI)**	**Category**	**Composite**	**Adjusted OR (95% CI)**	**Category**	**Composite**
**Overall**	948/5,891 (16)	NA	NA	NA	NA	NA	NA
Patient Characteristics							
**Patient sex**				0.02			0.01
Male	510/2,966 (17)	1.18 (1.03-1.36)	0.02		1.25 (1.05-1.48)	0.01	
Female	438/2,925 (15)	1 [Ref]	NA		1 [Ref]	NA	
**Age outlier for condition[a]**				0.15			0.25
Yes	107/589 (18)	1.18 (0.94-1.47)	0.15		1.17 (0.89-1.54)	0.25	
No	841/5,302 (16)	1 [Ref]			1 [Ref]	NA	
**Race/Ethnicity**				0.15			0.85
Non-Hispanic White	443/2,615 (17)	1.22 (1.03-1.44)	0.02		1.11 (0.89-1.38)	0.35	
Non-Hispanic Black	260/1,810 (14)	1 [Ref]	NA		1 [Ref]	NA	
Non-Hispanic Other	135/770 (18)	1.27 (1.01-1.59)	0.04		1.10 (0.83-1.47)	0.49	
Hispanic	103/648 (16)	1.13 (0.88-1.44)	0.35		0.98 (0.72-1.32)	0.88	
Unknown	7/110 (15)	1.02 (0.45-2.29)	0.97		0.98 (0.37-2.62)	0.97	
Admission Characteristics							
**Insurance**				<0.001			0.17
Medicaid	399/2,879 (14)	1 [Ref]	NA		1 [Ref]	NA	
Private	532/2,929 (18)	1.38 (1.20-1.59)	<0.001		1.17 (0.97-1.41)	0.10	
Unknown	17/83 (20)	1.60 (0.93-2.76)	0.09		1.47 (0.76-2.86)	0.25	
**Condition[b]**				<0.001			<0.001
**Year**				0.01			0.11
2015-2016[c]	183/1,363 (13)	1 [Ref]	NA		1 [Ref]	NA	
2017	197/1,212 (16)	1.25 (1.01-1.56)	0.04		1.06 (0.82-1.38)	0.66	

© 2023 Kern-Goldberger AS et al. *JAMA Network Open.*

2018	204/1,103 (18)	1.46 (1.18-1.82)	0.001		1.25 (0.96-1.62)	0.10	
2019	203/1,276 (16)	1.22 (0.98-1.51)	0.07		1.02 (0.79-1.32)	0.89	
2020	161/937 (17)	1.34 (1.06-1.68)	0.01		1.38 (1.04-1.84)	0.03	
Physician Characteristics							
**Attending experience (years)**				0.14			0.15
0-2	97/492 (20)	1.33 (1.04-1.69)	0.02		1.31 (0.96-1.78)	0.08	
3-10	433/2,771 (16)	1 [Ref]	NA		1 [Ref]	NA	
11-20	334/2,123 (16)	1.01 (0.86-1.18)	0.92		0.91 (0.75-1.11)	0.34	
21-40	84/505 (17)	1.08 (0.83-1.39)	0.57		1.08 (0.78-1.48)	0.64	
**Anxiety due to uncertainty quartile**				0.78			0.57
1st (bottom) quartile	371/2,365 (16)	1 [Ref]	NA		1 [Ref]	NA	
2nd quartile	176/1,032 (17)	1.11 (0.91-1.34)	0.32		1.01 (0.79-1.29)	0.92	
3rd quartile	265/1,633 (16)	1.04 (0.88-1.24)	0.65		1.11 (0.90-1.37)	0.33	
4th (top) quartile	136/861 (16)	1.01 (0.81-1.25)	0.94		0.91 (0.69-1.20)	0.52	
**Gender**				0.97			0.49
Male	270/1,677 (16)	1.00 (0.86-1.17)	0.98		0.92 (0.76-1.13)	0.43	
Female	661/4,114 (16)	1 [Ref]	NA		1 [Ref]	NA	
Prefer not to say	17/100 (17)	1.07 (0.63-1.81)	0.80		1.32 (0.67-2.62)	0.43	
Systems Characteristics							
**Inpatient hospital day**				<0.001			<0.001
1	372/935 (40)	1 [Ref]	NA		1 [Ref]	NA	
2-3	477/3,357 (14)	0.25 (0.21-0.30)	<0.001		0.32 (0.26-0.39)	<0.001	
4-6	83/1,365 (6)	0.10 (0.08-0.13)	<0.001		0.15 (0.11-0.20)	<0.001	
7-10	16/234 (7)	0.11 (0.07-0.19)	<0.001		0.21 (0.11-0.38)	<0.001	

© 2023 Kern-Goldberger AS et al. *JAMA Network Open.*

**Day of the week**				0.005			0.002
Mon/Tues	398/2,518 (16)	1 [Ref]	NA		1 [Ref]	NA	
Wed/Thurs/Fri	310/1,690 (18)	1.20 (1.02-1.41)	0.03		1.15 (0.95-1.40)	0.15	
Sat/Sun	240/1,683 (14)	0.89 (0.74-1.05)	0.17		0.78 (0.64-0.96)	0.02	
**Team category**				0.09			0.007
Resident/APP	661/3,967 (17)	1.14 (0.98-1.33)	0.09		1.31 (1.08-1.60)	0.007	
Frontline Hospitalist	287/1,924 (15)	1 [Ref]	NA		1 [Ref]	NA	
**Number of prior consultations during admission**				<0.001			<0.001
0	560/2,523 (22)	1 [Ref]	NA		1 [Ref]	NA	
1	281/2,001 (14)	0.45 (0.35-0.56)	<0.001		0.41 (0.32-0.52)	<0.001	
≥2	107/1,367 (8)	0.14 (0.08-0.22)	<0.001		0.10 (0.06-0.16)	<0.001	

Abbreviations: ref, Reference; APP, advanced practice practitioner; NA, not applicable
[a] ≤5th percentile or ≥95th percentile age for condition
[b] Consultation use by patient condition omitted for space
[c] Years combined in analysis as start of study period was October 1, 2015

eTable 13. Comparison of Single vs Multiple Consultation by Pediatric Hospitalists Among Patient-Days with ≥1 Consultation

		Unadjusted			Adjusted		
			P value			*P* value	
**Variable**	**No. of patient-days with multiple consultations, n/N (%)**	**OR (95% CI)**	**Category**	**Composite**	**Adjusted OR (95% CI)**	**Category**	**Composite**
**Overall**	154/1,108 (14)[d]	NA	NA	NA	NA	NA	NA
Patient Characteristics							
**Patient sex**				0.70			0.90
Male	81/600 (14)	0.93 (0.64-1.35)	0.70		1.03 (0.68-1.55)	0.90	
Female	73/508 (14)	1 [Ref]	NA		1 [Ref]	NA	
**Age outlier for condition[a]**				0.21			0.06
Yes	23/130 (18)	1.41 (0.82-2.42)	0.21		1.79 (0.97-3.31)	0.06	
No	131/978 (13)	1 [Ref]	NA		1 [Ref]	NA	
**Race/Ethnicity**				0.008			0.06
Non-Hispanic White	80/504 (16)	2.41 (1.37-4.23)	0.002		2.23 (1.20-4.13)	0.01	
Non-Hispanic Black	26/325 (8)	1 [Ref]	NA		1 [Ref]	NA	
Non-Hispanic Other	29/152 (19)	3.14 (1.55-6.37)	0.002		2.56 (1.19-5.53)	0.02	
Hispanic	19/119 (16)	2.41 (1.15-5.05)	0.02		2.01 (0.93-4.38)	0.08	
Unknown	0/8 (0)	1 (NA)	NA		1 (NA)	NA	
Admission Characteristics							
**Condition[b]**				0.03			0.052
**Insurance**				0.18			0.97
Medicaid	56/478 (12)	1 [Ref]	NA		1 [Ref]	NA	
Private	94/609 (15)	1.43 (0.96-2.13)	0.08		1.01 (0.64-1.60)	0.96	
Unknown	4/21 (19)	1.89 (0.53-6.71)	0.33		1.18 (0.29-4.76)	0.82	
**Year**				0.13			0.05
2015-2016[c]	27/226 (12)	1 [Ref]	NA		1 [Ref]	NA	
2017	26/222 (12)	0.99 (0.53-1.83)	0.97		0.53 (0.26-1.07)	0.08	
2018	35/243 (14)	1.27 (0.71-2.29)	0.42		0.88 (0.46-1.67)	0.70	
2019	45/234 (19)	1.85 (1.03-3.30)	0.04		1.67 (0.87-3.24)	0.13	

© 2023 Kern-Goldberger AS et al. *JAMA Network Open.*

2020	21/183 (11)	0.96 (0.50-1.85)	0.91		0.93 (0.45-1.91)	0.84	
Physician Characteristics							
**Attending experience (years)**				0.15			0.13
0-2	11/112 (10)	0.78 (0.38-1.63)	0.51		0.71 (0.31-1.63)	0.41	
3-10	60/492 (12)	1 [Ref]	NA		1 [Ref]	NA	
11-20	67/403 (17)	1.52 (0.99-2.34)	0.06		1.64 (0.99-2.70)	0.05	
21-40	16/102 (16)	1.35 (0.69-2.62)	0.38		1.64 (0.78-3.43)	0.19	
**Anxiety due to uncertainty quartile**				0.40			0.55
1st (bottom) quartile	62/445 (14)	1 [Ref]	NA		1 [Ref]	NA	
2nd quartile	30/197 (15)	1.13 (0.67-1.89)	0.66		1.22 (0.68-2.21)	0.54	
3rd quartile	46/303 (15)	1.12 (0.71-1.77)	0.62		1.10 (0.66-1.82)	0.72	
4th (top) quartile	16/163 (10)	0.65 (0.35-1.22)	0.18		0.67 (0.32-1.41)	0.29	
**Gender**				0.80			0.58
Male	45/319 (14)	1.01 (0.67-1.53)	0.96		0.77 (0.48-1.26)	0.30	
Female	107/767 (14)	1 [Ref]	NA		1 [Ref]	NA	
Prefer not to say	2/22 (9)	0.59 (0.12-2.84)	0.51		0.74 (0.11-4.78)	0.75	
Systems Characteristics							
**Inpatient hospital day**				0.02			0.57
1	78/427 (18)	1 [Ref]	NA		1 [Ref]	NA	
2-3	64/564 (11)	0.54 (0.35-0.82)	0.004		0.91 (0.59-1.42)	0.69	
4-6	10/98 (10)	0.46 (0.21-1.01)	0.05		1.27 (0.52-3.05)	0.60	
7-10	2/19 (11)	0.45 (0.09-2.38)	0.35		2.91 (0.47-17.93)	0.25	
**Day of the week**				0.26			0.57
Mon/Tues	67/475 (14)	1 [Ref]	NA		1 [Ref]	NA	
Wed/Thurs/Fri	42/357 (12)	0.80 (0.51-1.26)	0.34		0.80 (0.49-1.31)	0.38	
Sat/Sun	45/276 (16)	1.23 (0.78-1.95)	0.38		1.06 (0.64-1.74)	0.82	

**Team category**				0.14			0.10
Resident/APP	116/778 (15)	1.38 (0.90-2.13)	0.14		1.59 (0.91-2.77)	0.10	
Frontline Hospitalist	38/330 (12)	1 [Ref]	NA		1 [Ref]	NA	
**Number of prior consultations during admission**				0.003			<0.001
0	118/692 (17)	1 [Ref]			1 [Ref]	NA	
1	27/297 (9)	0.47 (0.28-0.79)	0.004		0.26 (0.14-0.46)	<0.001	
≥2	9/119 (8)	0.25 (0.08-0.78)	0.02		0.15 (0.04-0.53)	0.003	

Abbreviations: ref, Reference; APP, advanced practice practitioner; NA, not applicable
[a] ≤5th percentile or ≥95th percentile age for condition
[b] Consultation use by patient condition omitted for space
[c] Years combined in analysis as start of study period was October 1, 2015
[d] n=5 patient-days with multiple consultations attributed to different hospitalists excluded from this analysis

© 2023 Kern-Goldberger AS et al. *JAMA Network Open.*

eFigure 1. Methodology for Identifying Consultation Timing and Attending Attribution

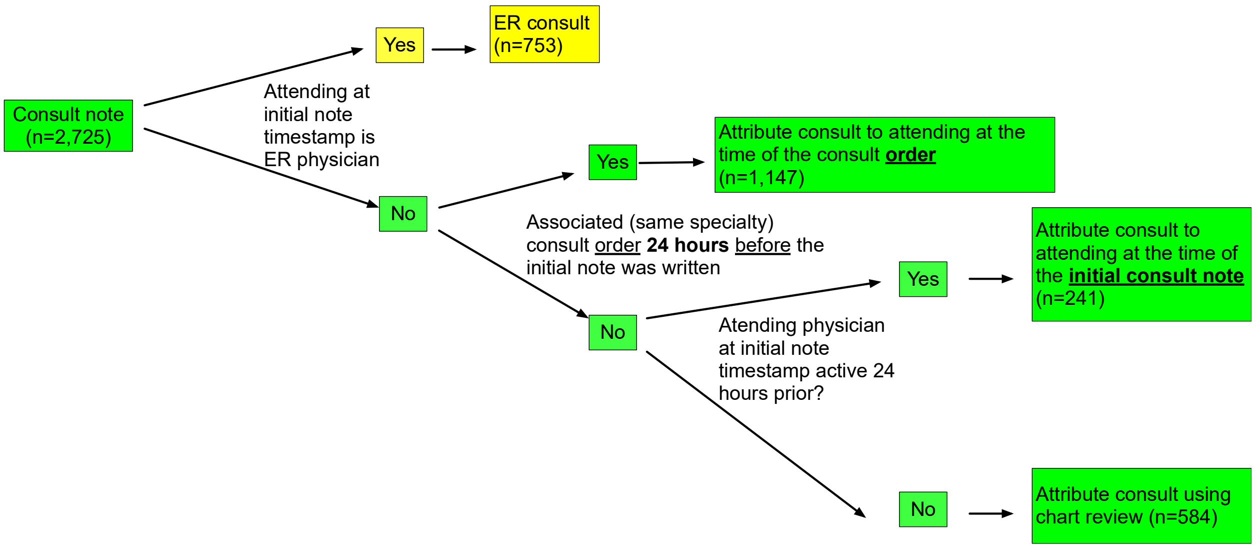

Consultations with a note initiation timestamp occurring during the time interval in which the active attending physician was a known Emergency Medicine attending (cross-checked with EHR specialty data) were attributed as “ER consultations.” For remaining consultation notes, we evaluated for a corresponding specialty consultation order during the 24 hours preceding the consultation note initiation timestamp. We attributed the consultation to the attending of record at the time of the consultation order. In the absence of a consultation order, we attributed consultations to the attending of record at the initial consultation note timestamp if the physician was also the attending of record in the 24 hours prior to this timestamp. For consultations that remained unattributed, we performed chart review and attributed consultations based on evaluation of clinical documentation and provider orders.

© 2023 Kern-Goldberger AS et al. *JAMA Network Open.*
