# Supplementary material for: Factors Associated With Inpatient Subspecialty Consultation Patterns Among Pediatric Hospitalists
Source: JAMA Netw Open. 2023 Mar 13;6(3):e232648. doi: 10.1001/jamanetworkopen.2023.2648 (PMC10011930; doi:10.1001/jamanetworkopen.2023.2648)
Supplement: Supplement 2. — Data Sharing Statement [file jamanetwopen-e232648-s002.pdf]

Data Sharing Statement

Kern-Goldberger. Factors Associated With Inpatient Subspecialty Consultation Patterns Among Pediatric Hospitalists. *JAMA Netw Open*. Published March 13, 2023. doi:10.1001/jamanetworkopen.2023.2648

Data
Data available: No
